# Green synthesis and application of ZnO nanoparticles for removing malathion and pyrene from aqueous solutions

**DOI:** 10.1038/s41598-026-61900-0

**Published:** 2026-07-20

**Authors:** Sabah Ibrahim, Shereen Shoeb, Belal Nodhy, Lubna A. Ibrahim

**Affiliations:** 1 Central Laboratory for Environmental Quality Monitoring, National Water Research Center, Shubra El Kheima, Egypt; 2 Water Management Research Institute (WMRI), National Water Research Center (NWRC), El-Qanater El-Khairia, 13621 Egypt

**Keywords:** Green, Kinetics, Malathion, Non-linear adsorption, Pyrene, NPs, Chemistry, Environmental sciences, Materials science, Nanoscience and technology

## Abstract

This study systematically investigates the green synthesis of ZnO nanoparticles using an *Artemisia* plant-extract-mediated sol–gel method and evaluates their performance for the simultaneous removal of malathion and Pyrene from aqueous solutions. The synthesized nanoparticles were thoroughly characterized by XRD, SEM, TEM, FTIR, XPS, and BET, revealing a highly crystalline, mesoporous structure with a surface area of 180 m^2^/g, pore diameters of 4–4.5 nm, and abundant surface hydroxyl groups and oxygen vacancies that facilitate adsorption. Batch experiments optimized pH (~ 7), adsorbent dosage (1 g/L), contact time (180 min), and initial pollutant concentrations. Non-linear kinetic modeling shows that adsorption follows the Pseudo-First-Order model (R^2^ ≥ 0.989, SSE < 10^–4^), indicating a physisorption process controlled by boundary layer mass transfer. Isotherm analysis fits the Langmuir model well (R^2^ ≥ 0.9588), giving maximum capacities at 298 K of 14.25 mg g^−1^ (malathion) and 26.74 mg g^−1^ (Pyrene), while Rₗ (0–1) and 1/n < 1 confirm favourable adsorption. Thermodynamic analysis demonstrated that malathion adsorption is endothermic and entropy-driven (ΔH° =  + 33.4 kJ/mol, ΔS° =  + 112.9 J/mol K), whereas Pyrene adsorption is exothermic and enthalpy-driven (ΔH° =  − 8.0 kJ/mol, ΔS° =  − 22.7 J/mol K), with negative ΔG° values confirming spontaneous adsorption under all studied conditions. The ΔH° values below 40 kJ mol^−1^ and ΔG° values in the range 0 to − 20 kJ mol^−1^ confirm the physical nature of the binding, and long-term sustainability. These findings indicate highlight the capability of green-synthesized ZnO nanoparticles to simultaneously remove structurally diverse organic pollutants under near-neutral conditions, addressing a key limitation in current adsorption systems. However, this study is limited to laboratory-scale conditions with limited evaluation of adsorbent regeneration and real wastewater applicability. Therefore, future work should focus on regeneration efficiency, long-term stability, and performance in real wastewater.

## Introduction

Environmental contamination by organophosphorus pesticides and polycyclic aromatic hydrocarbons (PAHs) represents a significant threat to both ecosystems and human health. The increasing co-occurrence of these contaminants in water systems highlights the urgent need for efficient and simultaneous removal strategies. Malathion (C_10_H_19_O_6_PS_2_) is a widely applied organophosphorus pesticide used extensively in agriculture, orchards, and vector control. Although effective, its environmental persistence and degradation into malaoxon, ametabolite with significantly higher toxicity compared to parent compound, pose serious ecological and public health concerns^1–5^. Documented toxicity effects include neurological dysfunction, respiratory distress, and potential fatality, particularly under prolonged exposure conditions^3–5^.

Various low-cost adsorbents have been explored for malathion removal, including hydroxyapatite-modified shells, apricot-stone activated carbon, bagasse fly ash, thermally treated sludge, drumstick peel powder, and chitosan alginate composites achieving promising removal efficiencies under optimized conditions^6–11^. However, these materials often suffer from limitations such as slow kinetics, narrow optimal pH ranges, insufficient recyclability, and reduced performance in multi-pollutant systems.

Pyrene (C_16_H_10_), a persistent polycyclic aromatic hydrocarbon, similarly poses serious environmental risks. Originating from incomplete combustion of fossil fuels, vehicular emissions, biomass burning, and tobacco smoke, pyrene is a ubiquitous pollutant present in water, soil, sediments, and food products^12–14^. Due to its lipophilicity, it bioaccumulates in aquatic organisms, causing oxidative stress, reproductive failure, and tissue damage, and is classified as a priority pollutant by the US EPA^15–17^. In humans, prolonged exposure is linked to possibly carcinogenic and mutagenic effects, organ failure, and severe irritation^13,18^. Existing removal methods such as alginate beads, peat adsorbents, green-synthesized iron oxide nanoparticles, activated carbon, coordination polymers, and MOFs (metal–organic frameworks) show limitation in cost, stability, low concentrations efficiency and stability^19–24^.

Zinc oxide nanoparticles (ZnO NPs) have emerged as a promising class of adsorbents due to their high surface area, strong reactivity, chemical stability, biocompatibility, and cost-effective green synthesis routes^6,20,25–27^. ZnO-based composites, including carbon-modified ZnO, graphene oxide/ZnO hybrids, and polyaniline/ZnO systems have shown enhanced adsorption of heavy metals and organic pollutants under various conditions^28–31^. Adsorption mechanisms are governed by complexation, ion exchange, electrostatic interactions, hydrogen bonding, and π–π stacking often allowing for efficient regeneration^32–35^.

Despite numerous studies on single-pollutant removal, no cost-effective and environmentally friendly adsorbent has been reported for simultaneous removal of both malathion and Pyrene under neutral conditions^32,35^. Most existing adsorbents display slow kinetics, limited pH tolerance, low reusability, and lack mechanistic insights. Addressing this gap is critical for sustainable water treatment strategies capable of targeting multiple contaminants simultaneously.

This study introduces green-synthesized ZnO nanoparticles as dual-function adsorbents for malathion and Pyrene. The novelty lies in the simultaneous removal of structurally distinct pollutants and comprehensive mechanistic characterization. The study aims to (1) determine the maximum adsorption capacity of ZnO for both contaminants, (2) elucidate the adsorption mechanisms through surface characterization, and (3) optimize operational parameters for effective pollutant removal.

## Material and methods

All adsorption experiments were conducted in triplicate, and the average values ± standard deviation were calculated by SPSS. The relative standard deviation (RSD) for all experiment was less than 5%, confirming good reproducibility and reliability of the experimental data.

### Chemicals and reagents

All chemicals used in this study were of analytical grade and used as received. Malathion and pyrene stock solutions were prepared in distilled water. Zinc acetate dihydrate (Zn(CH₃COO)₂·2H₂O, purity > 99%, Sigma-Aldrich, USA), malathion (analytical standard grade, Sigma-Aldrich), and pyrene (≥ 98% purity, Sigma-Aldrich) were used as received. Ethanol (99.5%, Vetec Química Fina, Brazil) was used as solvent. Triethylamine (TEA, ≥ 99% purity, Sigma-Aldrich) was used as a base catalyst and complexing agent to control the hydrolysis and condensation reactions during ZnO nanoparticle synthesis. Deionized water was produced using a Thermo Scientific Smart@Pure 6 UV system. Sodium chloride (NaCl, ≥ 99%, Sigma-Aldrich) was used for pHpzc determination. All chemicals were used without further purification to ensure experimental consistency.

### Preparation of *Artemisia* plant extract

*Artemisia absinthium* (wormwood) leaves were collected from local cultivated gardens in Shubra El Kheima, Egypt, during the spring season. Leaves of *Artemisia* (wormwood) were collected, washed with distilled water to remove dust and impurities, and air-dried at room temperature for 72 h, and finely ground. Approximately 10 g of the powder was boiled in 100 mL of distilled water at 50 °C for 60 min using a magnetic hot plate using a magnetic stirrer. The extraction conditions (50 °C, 60 min) were selected to preserve thermolabile bioactive phytochemicals, including polyphenols, flavonoids, and terpenoids, responsible for nanoparticle stabilization and capping^36^. After that, the mixture was cooled to room temperature and filtered using Whatman No. 1 filter paper to obtain a clear extract. The filtrate was stored at 4 °C and used within 48 h as a green stabilizing and capping agent in the synthesis of ZnO nanoparticles use^36^.

### Synthesis of zinc oxide nanoparticles

ZnO nanoparticles were synthesized using a sol–gel method in which *Artemisia* plant extract as a green served as a green capping, enabling precise control over particle size and morphology^35^. Briefly, 7.68 g of zinc acetate was dissolved in 35 mL of ethanol to prepare a 0.5 M solution and stirred at 60 °C. TEA was then added in a 1:1 molar ratio with Zn^2^⁺, and the mixture was stirred at 60 °C for 1 h to obtain a homogeneous sol. Subsequently, 20 mL of the filtered *Artemisia* extract was added dropwise under continuous stirring, and the mixture was further stirred for 30 min to allow phytochemical adsorption onto forming ZnO nuclei. The resultant solution was aged at room temperature for 1 h, transferred to a 50 mL Teflon-lined autoclave, and heated at 150 °C for 18 h. The white precipitate was collected, washed three times with 30% (v/v) ethanol/deionized water by centrifugation at 4000 rpm for 4 min, and dried in an oven at 60 °C overnight. Hydrothermal treatment ensures high crystallinity and controlled morphology of ZnO nanoparticles.

### Preparation of malathion and pyrene stock solutions

Stock solutions of malathion and pyrene were prepared at 1000 µg L^−1^^34^. Pyrene stock was dissolved in ethanol and stored at 278 K. Working solutions at 50–500 µg L^−1^ (malathion) and 50–200 µg L^−1^ (pyrene) were prepared daily by diluting the stock solutions with deionized water to achieve the desired concentrations.

for adsorption experiments.

### Zero-point charge (pH_p_zc)

**Fig. 1:**
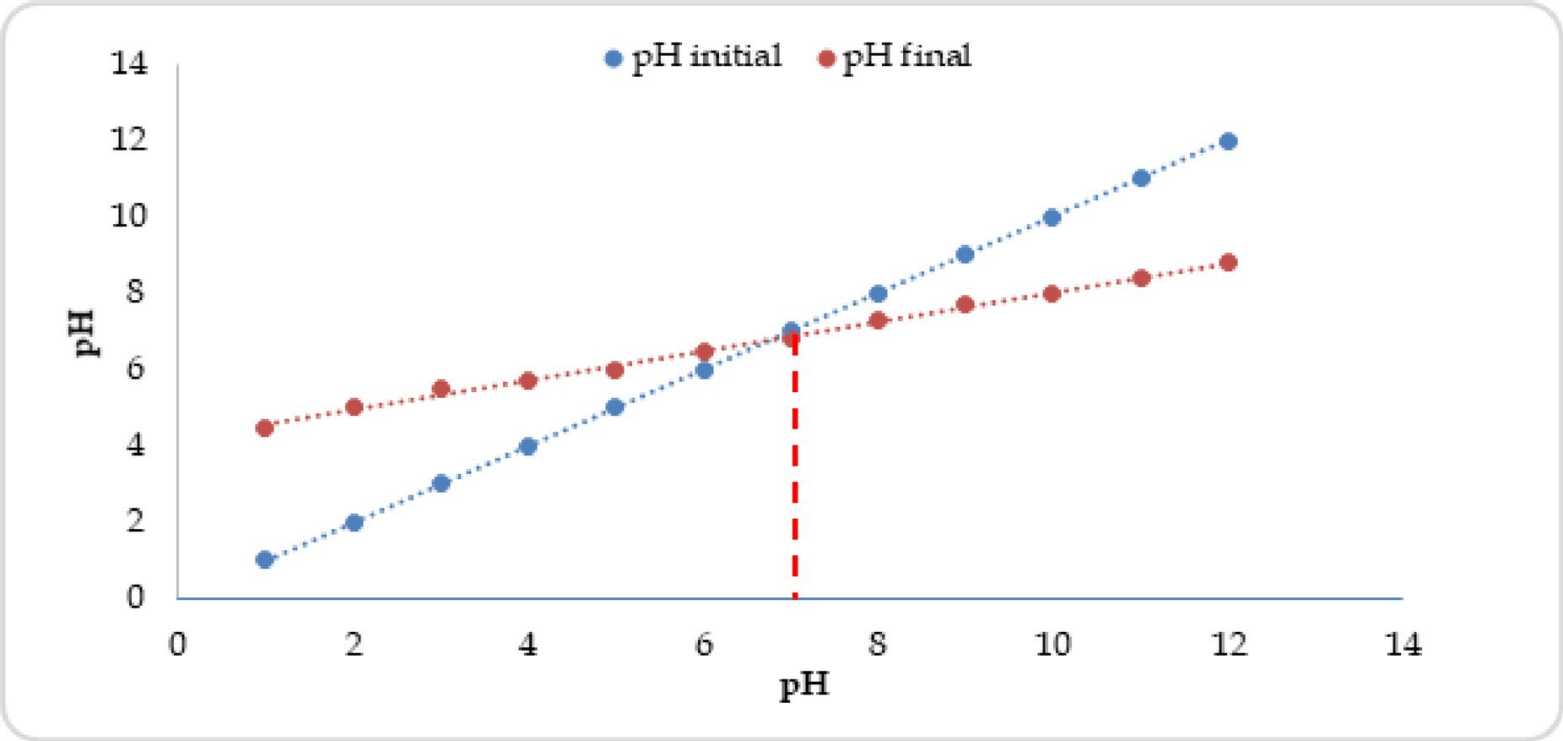
The point of zero charge (pHpzc) of zinc oxide (ZnO) nanoparticles by comparing the initial solution pH with the final pH after equilibrium.

The zero-point charge (pH_p_zc) of ZnO nanoparticles was determined to by the pH drift method understand surface charge behavior, which is critical for adsorption applications^32^. A 0.2 g sample of ZnO was added to 50 mL of 0.1 M NaCl solutions adjusted to initial pH 2–11. After agitation at 150 rpm for 48 h and centrifugation, the difference ΔpH (= pHinitial − pHfinal) was plotted against pH_initial_, and the pHpzc corresponds to ΔpH = 0. Figure 1 illustrates the pHpzc was ~ 6.8–7.0, consistent with reported values for ZnO (6–8)^25,35^. At pH < pH_p_zc the ZnO surface is positively charged; at pH > pH_p_zc it is negatively charged, modulating the adsorption of anionic and cationic species respectively. The near-neutral pH_p_zc observed here supports these findings, indicating that ZnO nanoparticles can effectively interact with both anionic and cationic contaminants through simple pH adjustment, thereby confirming their suitability for environmental remediation and wastewater treatment applications.

### Characterization techniques

The synthesized ZnO nanoparticles were characterized using multiple techniques. Crystallinity was determined using X-ray diffraction (XRD, Bruker D8, Cu Kα radiation, λ = 1.54 Å). Crystallite size was calculated using the Scherrer equation D = Kλ/(β cos θ) with FWHM determined by Gaussian peak fitting. Surface morphology was examined by SEM (Quanta 250, FEI) with EDX, and TEM (JEOL-JEM-2100 FS, 200 kV). Functional groups were identified by FTIR (Bruker VERTEX 80 V, 400–4000 cm^−1^). Surface composition was analyzed using X-ray photoelectron spectroscopy (XPS, K-ALPHA^+^, Thermo Fisher Scientific). Textural properties were determined from N₂ adsorption–desorption isotherms (Micromeritics ASAP 2020). Concentrations were quantified using GC–MS (Agilent 7890A).

### Adsorption studies

Batch adsorption experiments were performed to evaluate the removal efficiency of ZnO nanoparticles for malathion and pyrene. All experiments were conducted in 250 mL Erlenmeyer flasks containing 100 mL of aqueous solution. Initial concentrations of malathion and pyrene were varied from 100 to 500 µg L^−1^, and 50–200 µg L^−1^, respectively. Adsorbent dosage ranged from 0.1 to 1.0 g/L (equivalent to 0.01–0.10 g per 100 mL). Flasks were agitated on a thermal shaker at 210 rpm and 25 °C for predetermined times up to 180 min. Effects of pH (3.0, 5.0, 7.0, 9.0, 11.0), contact time (20–180 min), adsorbent dosage (0.1–1.0 g/L), and initial contaminant concentration (50–150 µg L^−1^) were systematically studied to optimize adsorption conditions. Percentage removal efficiency (%RE) was calculated using Eq. 1:

1 $$\mathrm{\%}\mathrm{R}\mathrm{E}=\frac{{\mathrm{C}}_{\mathrm{o}}-{\mathrm{C}}_{\mathrm{e}}}{{\mathrm{C}}_{0}}\times 100$$

where C_0_ and C_e_ are the initial and equilibrium concentrations of malathion and pyrene in the solution (mg/L) (µg L^−1^), respectively. The equilibrium adsorption capacity (qe, mg g^−1^) was calculated using Eq. 2:

2 $$\mathrm{q}\mathrm{e}=\frac{({\mathrm{C}}_{\mathrm{i}}-{\mathrm{C}}_{\mathrm{e}})}{\mathrm{m}}\times \mathrm{V}$$

where Ci and Ce are the initial and equilibrium concentrations (mg L^−1^), V is solution volume (L), and m is adsorbent mass (g), yielding qe in mg g^−1^.

### Adsorption kinetics studies

ZnO NPs (0.10 g) were added to 100 mL of solution containing 100 µg L^−1^ malathion and 50 µg L^−1^ pyrene and agitated at 25 °C. Samples were withdrawn at 20, 40, 60, 90, 120, 150, and 180 min. Kinetics were modeled using non-linear Pseudo-first-order PFO (Eq. 3), Pseudo-second-order PSO (Eq. 4), and Elovich (Eq. 5) models^17,28,32^ to determine the controlling adsorption mechanisms and rate constants, Table 1.

**Table 1:** Non-linear forms of adsorption isotherms and definitions of parameters.

Model	Non-Linear Form Equations	Parameters
Pseudo-first-order PFO	${\text{qt }} = {\text{ qe}}(1 - {\text{ }}e^{{( - k_{2} t)}} )$	where k₁ (min^−1^) and k₂ (g mg^−1^ min^−1^) are PFO and PSO rate constants; qe and qt (mg g^−1^) are adsorption capacities at equilibrium and time t; α (mg g^−1^ min^−1^) and β (g mg^−1^) are Elovich constants
Pseudo-second-order PSO	${\text{qt }} = {\text{ k}}_{{2}} {\mathrm{qe}}^{{2}} {\mathrm{t}}/\left( {{1} + {\mathrm{k}}_{{2}} {\mathrm{qet}}} \right)$	
Elovich	${\text{qt }} = \, \left( {{1}/\beta } \right){\mathrm{ln}}\left( {{1} + \alpha \beta {\mathrm{t}}} \right)$	
Langmuir	$${\text{qe }} = \, \left( {{\text{Qmax }}\cdot{\text{ KL }}\cdot{\text{ Ce}}} \right)/\left( {{1 } + {\text{ KL}}\cdot{\text{ Ce}}} \right)$$$${\text{RL }} = { 1 }/ \, \left( {{1 } + {\text{ KL }}\cdot{\text{ C}}0} \right)$$	qe = adsorption capacity (mg/g); Ce = equilibrium concentration (mg/L); Qmax = maximum adsorption capacity (mg/g); KL = Langmuir constant (L/mg); C0 = initial concentration (mg/L); RL = separation factor
Freundlich	${\text{qe }} = {\text{ KF }}\cdot{\text{ Ce}}^{{({1}/{\mathrm{n}})}}$	KF = Freundlich capacity factor [(mg/g)(L/mg)^(1/n)]; 1/n = heterogeneity factor
Temkin	${\text{qe }} = {\text{ B }}\cdot{\text{ ln}}\left( {{\text{AT }}\cdot{\text{ Ce}}} \right)$	AT = equilibrium binding constant (L/g); B = heat of sorption constant (J/mol)
Redlich–Peterson	${\text{qe }} = \, \left( {{\text{KR }}\cdot{\text{ Ce}}} \right) \, / \, \left( {{1 } + {\text{ aR }}\cdot{\text{ Ce}}^{\beta } } \right)$	KR (L/g), aR (L/mg^β) = constants; β = heterogeneity exponent (0 ≤ β ≤ 1)
Dubinin–Radushkevich	${\text{qe }} = {\text{ qm }} \cdot {\text{ }}\exp \left( { - {\text{Kad }} \cdot \varepsilon } \right)$ $\varepsilon \, = {\text{ RT }}\cdot{\text{ ln}}\left( {{1 } + { 1}/{\mathrm{Ce}}} \right)$ ${\text{E }} = { 1}/\surd \left( {{\mathrm{2Kad}}} \right)$	qm = saturation capacity (mg/g); Kad = D-R constant (mol^2^/J^2^); ε = Polanyi potential (J/mol); E = mean free energy (kJ/mol)

### Adsorption isotherms studies

Equilibrium studies used 100 mL solutions with varying initial concentrations of malathion (50–500 µg L^−1^) and pyrene (50–200 µg L^−1^) with 0.10 g ZnO NPs at 298–338 K for 3 h. Residual concentrations were measured and data fitted to Langmuir (Eqs. 6 &7), Freundlich (Eq. 8)^29^, Temkin (Eq. 9)^14^ Redlich–Peterson (Eq. 10), and Dubinin–Radushkevich (Eqs. 11–13) models. Table 1 presents the five nonlinear adsorption isotherm models employed in this study, together with their mathematical forms (Eqs. 6–13) and the associated parameters. Nonlinear regression was applied directly to avoid the error distribution distortion inherent in linearization transformations.

#### Thermodynamic studies

Thermodynamic parameters were determined at five temperatures (298, 308, 318, 328, and 338 K) using 0.10 g ZnO NPs in 100 mL solution at pH ~ 7, initial malathion 100 µg L^−1^ and pyrene 50 µg L^−1^, contact time 180 min, and agitation at 210 rpm. The Van’t Hoff equation (Eq. 14) was used to derive ΔH° and ΔS° from a plot of ln KL vs. 1/T, and ΔG° was calculated from Eq. 15.

14 $${\mathrm{lnK}}_{\mathrm{L}}= \frac{{-\Delta \mathrm{H}}^{^\circ }}{\mathrm{R}\mathrm{T}}+ \frac{{\Delta \mathrm{S}}^{^\circ }}{\mathrm{R}}$$

15 $$\Delta \mathrm{G}^\circ = \Delta \mathrm{H}^\circ -\mathrm{T}\Delta \mathrm{S}^\circ$$

## Results and discussion

### Characterization of ZnO nanoparticles

#### EDX, TEM, and SEM analyses

**Fig. 2:**
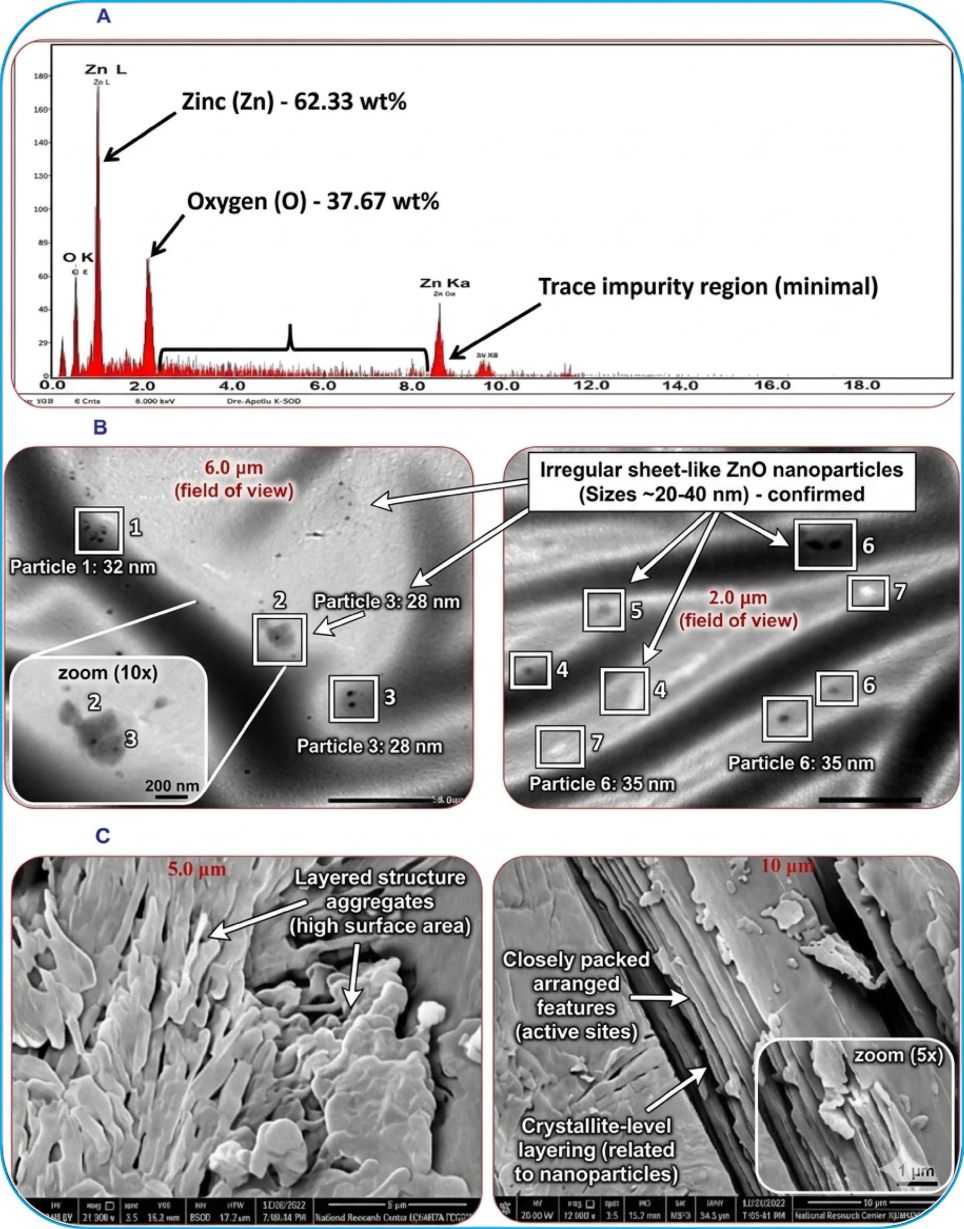
(**A**) Energy Dispersive X-ray of Zinc Oxide nanoparticles; while (**B**) is Transmission Electron Microscopy of Zinc Oxide nanoparticles, and (**C**) is Scanning Electron Microscopy of Zinc Oxide nanoparticles.

EDX analysis (Fig. 2A and Table 2) confirmed Zn at 62.33 wt% and O at 37.67 wt%, indicating a nearly stoichiometric ZnO composition consistent with prior reports^34,35^. The EDX spectrum demonstrates the elemental composition of the ZnO nanoparticles, confirming the presence of prominent Zn L, Zn Kα, and Zn Kβ peaks, along with the O K peak**, **Fig. 2A. Trace elements, if any, are minimal, indicating a high purity of the synthesized material. These results are consistent with prior studies by^34,35^, validating the successful synthesis of ZnO nanoparticles. The elemental purity supports their potential application as efficient adsorbents, as impurities can hinder adsorption efficiency.

**Table 2:** Elemental composition of Zinc Oxide nanoparticles.

Element	Weight %	Atomic %	Net Int	Error %
O K	37.67	71.18	28.25	4.44
Zn K	62.33	28.82	31.8	3.8

TEM analysis (Fig. 2B) revealed nanoparticles with an irregular, sheet-like morphology; scale bars in the micrographs confirm particle dimensions in the 20–40 nm range, consistent with the XRD-derived crystallite size of 25.8 nm. The uniformity of particle size and shape is essential for maximizing surface area and enhancing adsorption efficiency. Similar observations were reported by Mohana and Renjanadevi^34^, who noted that high-crystallinity ZnO nanoparticles often exhibit layered or rod-like structures.

SEM analysis (Fig. 2C) highlighted layered, elongated structures with closely packed arrangements, providing a high surface area and numerous active sites. The combined morphology supports both physisorption and chemisorption of malathion and pyrene^34,35^. The surface topology observed here is in agreement with Al-Darwesh et al.^35^.

#### FTIR analysis

**Fig. 3:**
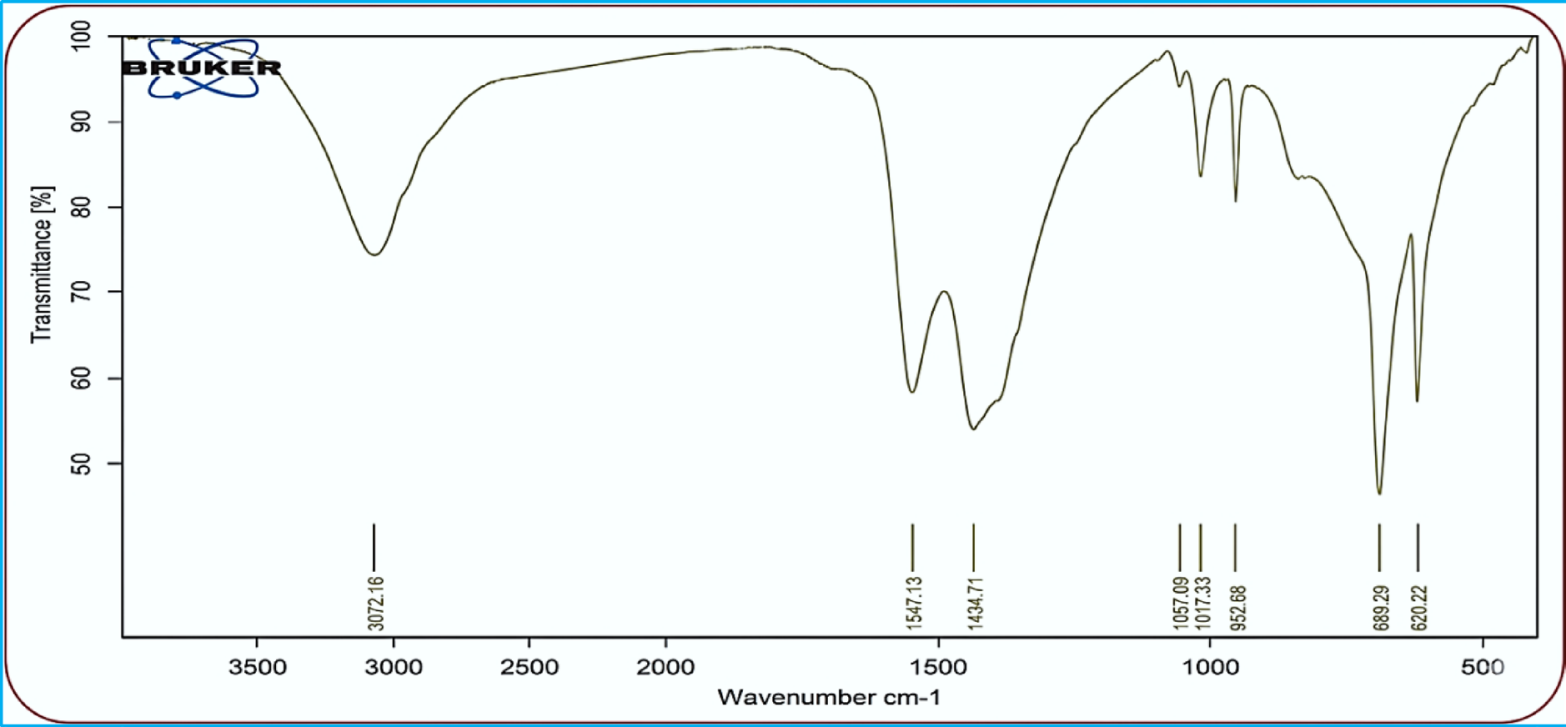
Fourier Transform Infrared Spectroscopy of Zinc Oxide nanoparticles.

The FTIR spectrum (Fig. 3) shows band centred at ≈ 3372 cm^−1^ is attributed to the O–H stretching vibrations of surface hydroxyl groups and physically adsorbed water molecules^41–43^. These hydroxyl groups play a critical role in adsorption processes, as they facilitate hydrogen bonding and electrostatic interactions with polar contaminants such as malathion^8,32^. The absorption bands observed at ≈ 1545 and 1427 cm^−1^ are associated with O–H bending vibrations and weak residual organic moieties originating from the plant extract used during green synthesis. These bands do not indicate impurity phases, but rather reflect surface functionalization that can enhance adsorption by increasing surface polarity and active site availability, as reported in earlier studies on green-synthesized ZnO nanoparticles^41,42^.

Bands appearing in the region ≈ 1057–952 cm^−1^ are assigned to Zn–O–H, surface C–O vibrational modes, which are commonly observed in ZnO nanoparticles with chemically active surfaces. Most importantly, the strong absorption bands below 600 cm^−1^ (≈ 520–450 cm^−1^) correspond to Zn–O lattice vibrations below 600 cm^−1^, in excellent agreement with authenticated ZnO FTIR spectra^41–44^.

#### XRD analysis

**Fig. 4:**
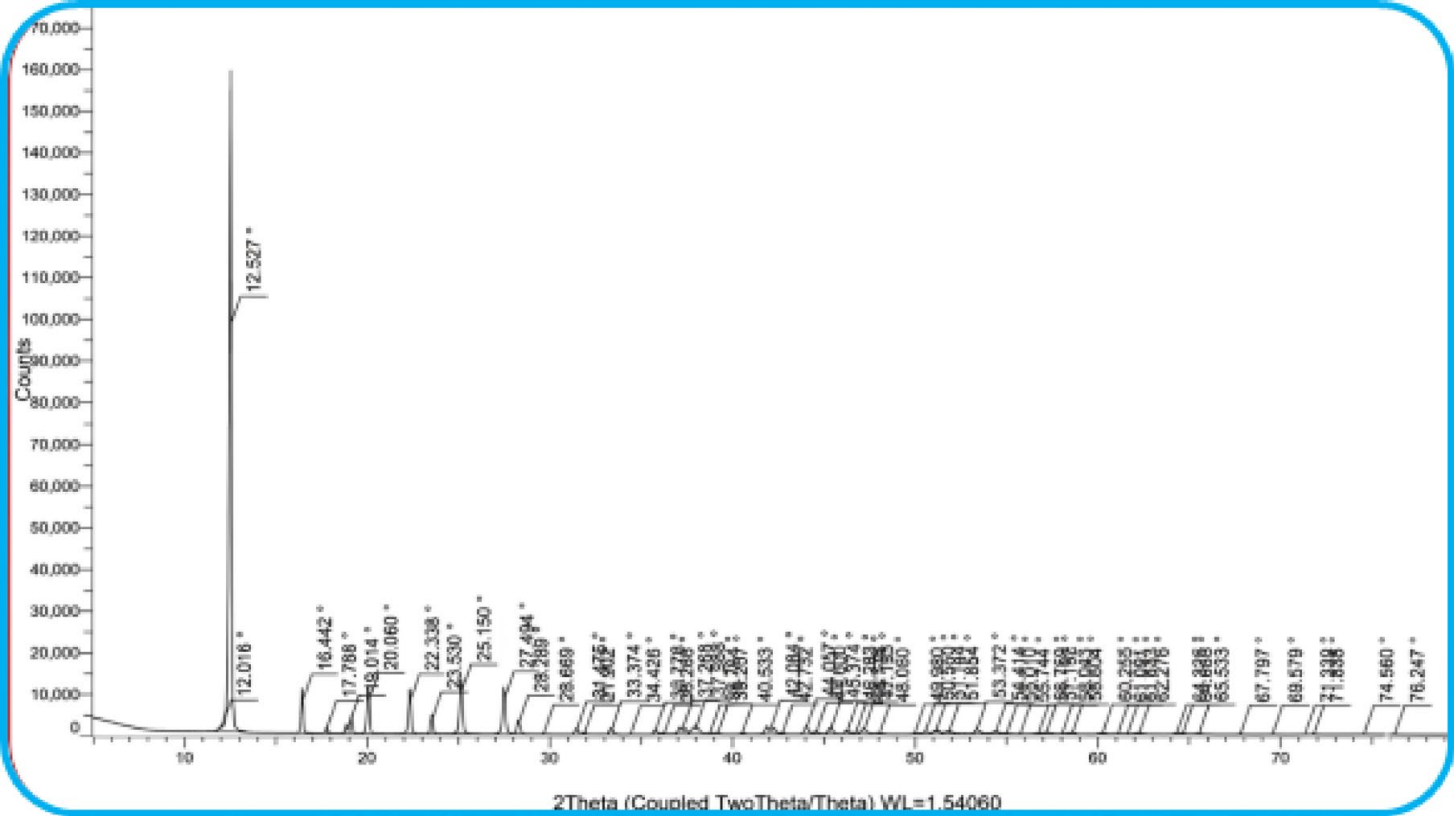
X-ray diffraction of Zinc Oxide nanoparticles.

The XRD pattern (Fig. 4) exhibits peaks at 2θ ≈ 31.7°, 34.4°, 36.2°, 47.5°, 56.6°, 62.8°, and 67.9°, indexed to the hexagonal wurtzite ZnO structure (JCPDS 36–1451). The absence of additional sharp peaks indicates high phase purity, with no detectable crystalline Zn(OH)₂ or metallic Zn. Notably, a broad, low-intensity peak at ~ 12.5° is observed, which we attribute to the presence of residual organic capping species derived from the *Artemisia* extract rather than crystalline impurity phases. This is consistent with previous reports on plant-mediated ZnO synthesis, where the organic template-nanoparticle interface manifests as low-angle reflections^47,48^. This attribution is further supported by our FTIR results, Fig. 3, which demonstrate characteristic vibrational modes of polyphenolic compounds from the *Artemisia* extract; specifically, the absorption bands at ~ 3072 cm^−1^ (aromatic C-H stretching) and ~ 1547 and ~ 1434 cm^−1^ (C = C aromatic ring vibrations and carboxylate groups) confirm the presence of residual organic capping layers on the ZnO surface, consistent with previous observations in plant-mediated ZnO systems^43,45–47^, supporting our observation of residual capping layers on the nanostructure surface. This observation is consistent with green synthesis protocols^47,48^. Crystallite size was calculated using the Scherrer equation with FWHM determined by Gaussian peak fitting from the (101) reflection, yielding D = 25.8 nm.

#### XPS analysis

**Fig. 5:**
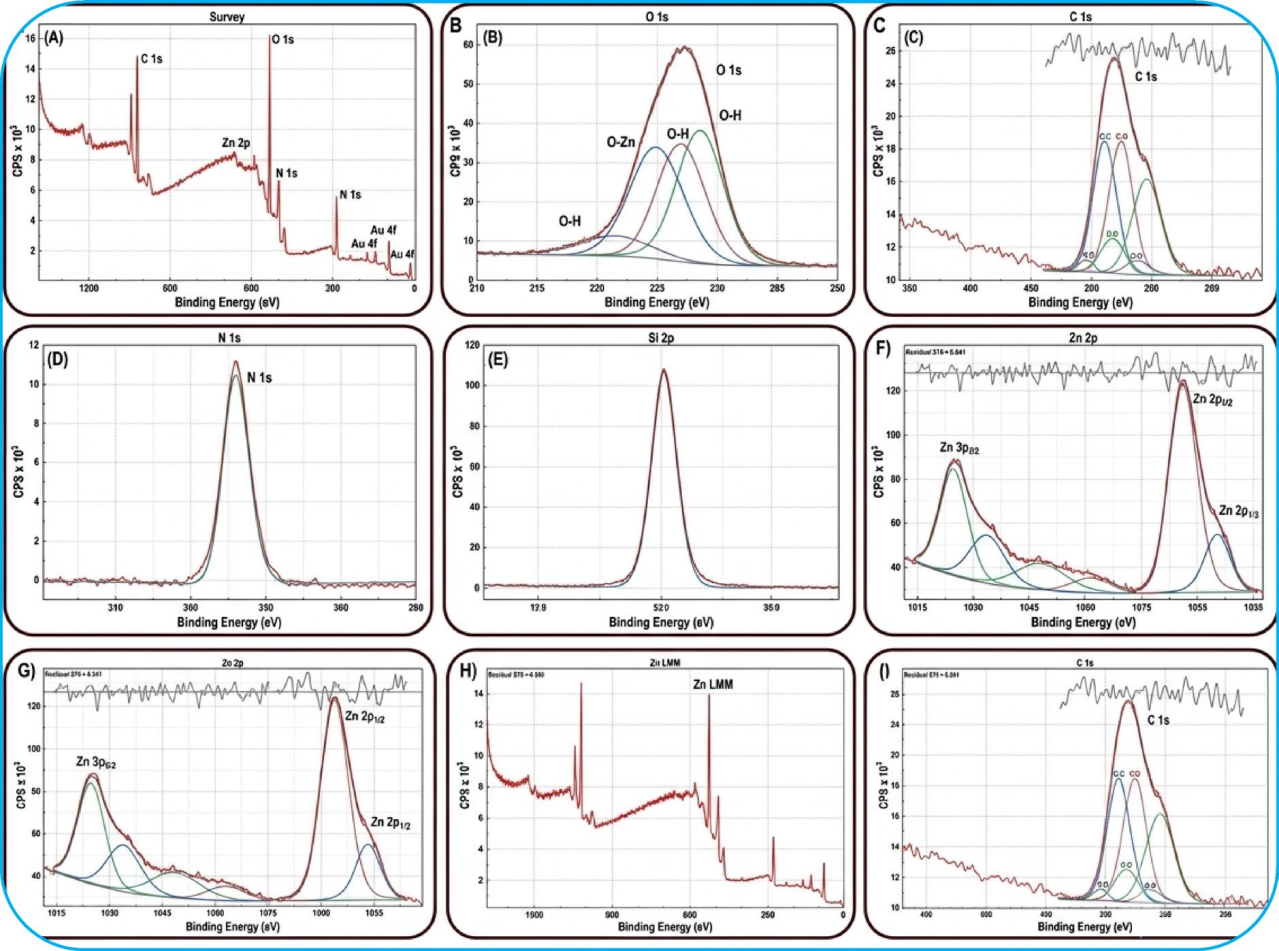
X-ray Photoelectron Spectroscopy of Zinc Oxide nanoparticles.

XPS survey spectra confirmed Zn, O, and C elements (Fig. 5). The C 1 s peak arises from adventitious carbon contamination. High-resolution Zn 2p spectra showed Zn 2p_3/2_ and Zn 2p_1/2_ peaks confirming Zn^2^⁺ in the ZnO lattice^32,34^. Shifts in Zn 2p and O 1 s binding energies are attributed to variations in surface oxygen vacancy (VO) density and chemisorbed hydroxyl group concentration arising from the sol–gel/hydrothermal synthesis conditions^49^. Deconvolution of the asymmetric O 1 s peak revealed contributions from lattice oxygen (OL), oxygen vacancies (VO), and surface hydroxyl/adsorbed water species. These surface features serve as active Lewis acid sites for pollutant adsorption^32,35^. FTIR analysis confirmed the functional groups in the range of 400–4000 cm^−1^. Characteristic Zn–O stretching vibrations were observed in the range of 400–600 cm^−1^, which is in excellent agreement with authentic reported spectra of ZnO nanomaterials. Specific bands at 3372 cm^−1^ and 1057–952 cm^−1^ are assigned to O–H and Zn–O–H vibrations, respectively.

#### Textural properties and surface area analysis

The textural properties of the synthesized ZnO nanoparticles were investigated using N₂ adsorption–desorption analysis. The corresponding BET and BJH parameters are summarized in Table 3. ZnO nanoparticles exhibited a relatively high specific surface area (SBET) = 180 m^2^ g^−1^, BET constant C = 345.48, total pore volume 0.085 cm^3^ g^−1^, and average pore diameters of 4.04 nm (BET) and 4.55 nm (BJH), confirming a predominantly mesoporous structure facilitating efficient mass transfer , which is advantageous for adsorption applications. The BJH pore volume (0.079 cm^3^ g^−1^) closely agrees with the BET-derived volume, indicating good consistency between the two methods.

**Table 3:** Textural properties of ZnO nanoparticles.

Parameter	Value (ZnO Nps)	Unit
Crystal Structure	Hexagonal Wurtzite	–
Crystallite Size	25.8	nm
BET surface area (SBET)	180	m^2^ g^−1^
Monolayer volume (Vm)	0.64	cm^3^(STP) g^−1^
BET constant (C)	345.48	–
Total pore volume (P/P₀ = 0.99)	0.085	cm^3^ g^−1^
Average pore diameter (BET)	4.0	nm
Average pore diameter (BJH)	4.5	nm
Median pore diameter (BJH)	2.93	nm
BJH pore volume	0.079	cm^3^ g^−1^

The monolayer adsorption volume (Vm = 0.64 cm^3^ STP g^−1^) and the high BET constant (C = 345.48) indicate strong interactions between the adsorbate molecules and the ZnO surface, suggesting a chemically active surface with high adsorption affinity. These characteristics are commonly associated with effective adsorbents for organic contaminants.

Overall, the combination of high surface area and well-developed mesoporosity ensures efficient mass transfer and abundant active sites, strongly contributing to the enhanced adsorption performance of ZnO nanoparticles toward malathion and pyrene, as observed in subsequent kinetic and isotherm studies.

### Effects of key experimental parameters on adsorption

#### Effect of Initial pH

**Fig. 6:**
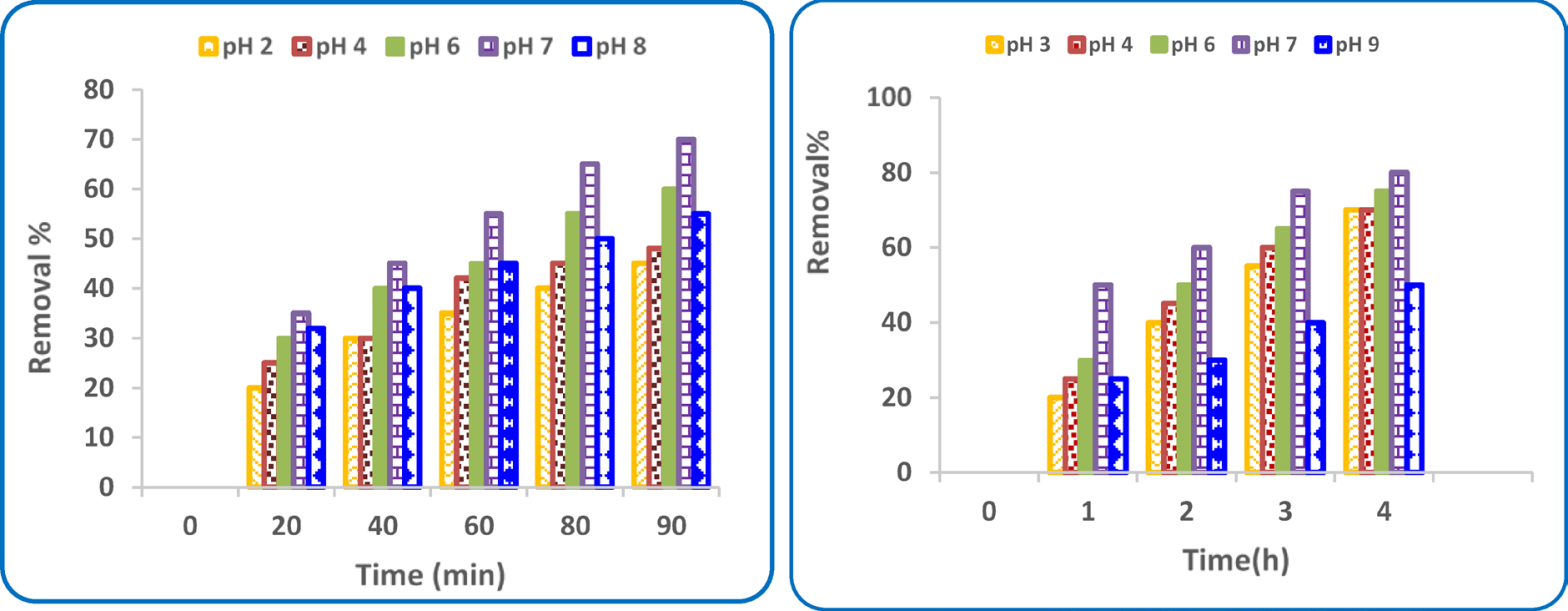
Effect of pH, for A- malathion and B- Pyrene by ZnO.

Initial pH critically influences adsorption of malathion and pyrene onto ZnO nanoparticles (Fig. 6). At pH < pHpzc the ZnO surface is positively charged, favoring anionic pollutant species. At pH > pHpzc the surface is negatively charged. Maximum adsorption efficiency for both pollutants was observed at neutral pH ~ 7 (optimal pH), where the near-neutral surface charge minimizes competitive ion effects and maximizes active site availability for hydrogen bonding, electrostatic interactions, and π–π/hydrophobic interactions. For malathion (contains phosphate/carboxylate groups), near-neutral pH avoids excessive H⁺ competition (pH < 5) and hydroxide competition (pH > 9). For pyrene (non-ionic, hydrophobic), removal remained consistently high (> 90%) across pH 5.0–8.0 due to the dominance of non-ionic hydrophobic interactions and stacking. Adsorption is largely independent of electrostatic effects and governed primarily by π–π stacking and hydrophobic interactions, consistent with Kumar et al.^5^ and Barreca et al.^19^.

#### Effect of contact time

**Fig. 7:**
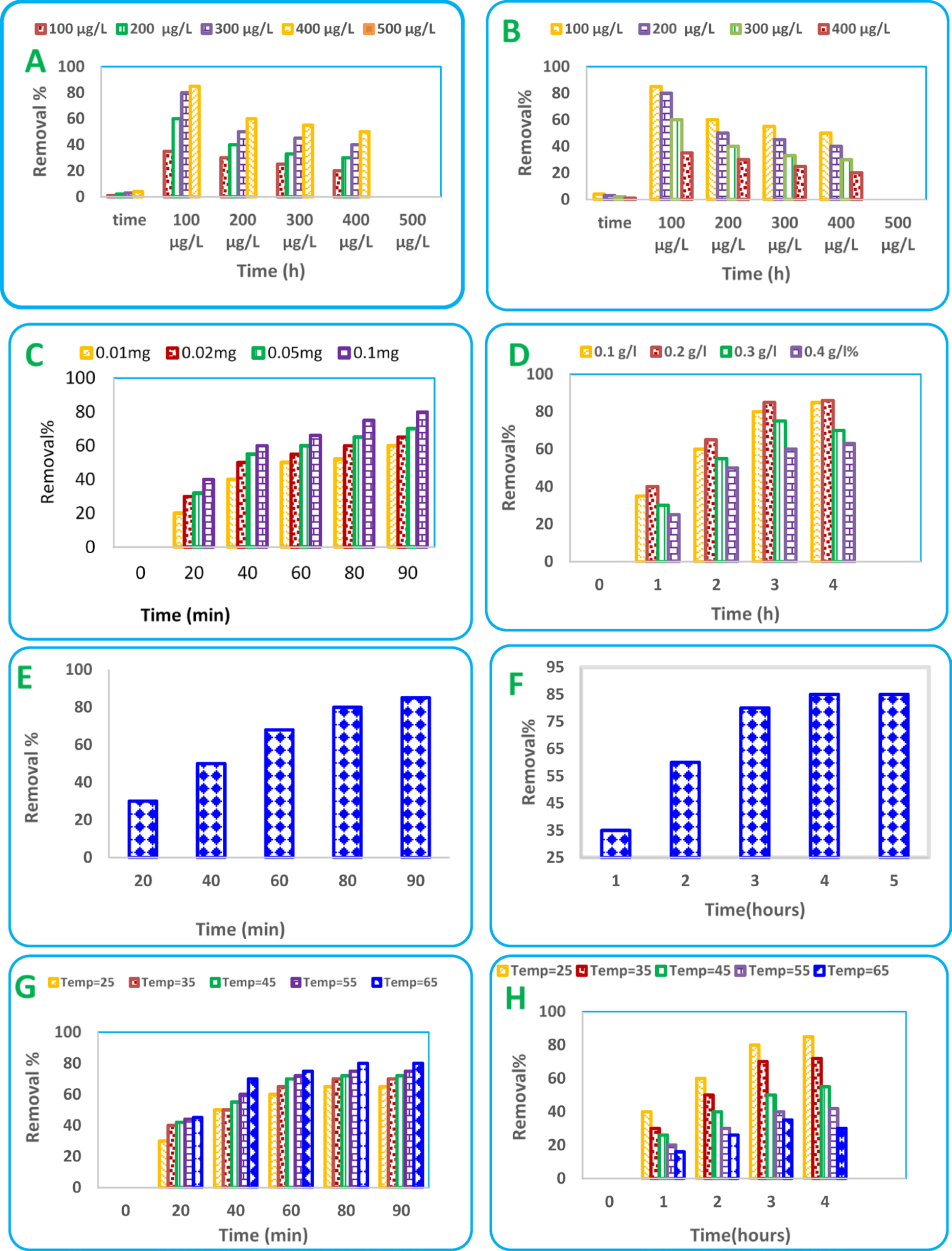
Effects of ZnO on malathion and pyrene: exposure time (**A**, **B**), dose (**C**, **D**), initial concentration (**E**, **F**) and temperature (**G**, **H**).

Contact time experiments, Fig. 7A & B, indicated rapid initial adsorption occurred within the first 60 min, corresponding to the occupation of easily accessible surface sites. Adsorption gradually approached equilibrium around 180 min, after which removal rates plateaued. The kinetic profile suggests that adsorption involves surface chemisorption and intraparticle diffusion, consistent with pseudo-second-order kinetic behavior. The rapid initial uptake followed by equilibrium is in agreement with Hassan et al.^21^ and Girardello et al.^20^, where ZnO and similar nanoparticles displayed fast adsorption for PAHs and organophosphate pesticides due to high surface area and porous structures.

#### Effect of adsorbent dosage

The adsorbent dosage ranged from 0.1 to 10 g/L (equivalent to 0.1–1.0 g per 100 mL), Fig. 7C& D. Adsorption efficiency increased with increasing ZnO dosage initially due to a higher number of available active sites, with marginal improvement beyond 0.5 g L^−1^. Optimal dosage for both malathion and pyrene were determined as 0.1 g per 100 mL solution (1 g/L), balancing removal efficiency and material economy. Similar behavior is noted by Suo et al.^1^ and Tchounwou et al.^2^, where increasing adsorbent dose improves removal until saturation occurs. This confirms that adsorbent dosage optimization is essential for practical applications to prevent waste and cost inefficiency.

#### Effect of initial concentration

Initial concentration was studied over 100–400 µg L^−1^ (malathion) and 50–150 µg L^−1^ (pyrene) (Fig. 7E and F). Adsorption capacity qe increased with increasing initial concentration due to the greater mass-transfer driving force. However, percentage removal decreased at higher concentrations as available active sites became insufficient relative to the pollutant load. These trends indicate that adsorption is both concentration- and surface-limited, consistent with Langmuir-type monolayer behavior observed in isotherm studies^26,28^.

Optimal pH: ~ 7 (neutral), maximizing active site availability and minimizing competitive ion effects. Optimal adsorbent dosage: 0.1 g per 100 mL (1 g/L) solution, balancing efficiency and economy. Optimal contact time: 180 min, ensuring equilibrium is reached without unnecessary delay. Initial concentration influence: Higher concentrations increase adsorption capacity but reduce percentage removal. These results align with existing literature^1,5,19,21,26–28^, demonstrating that ZnO nanoparticles are effective adsorbents when key experimental conditions are optimized.

#### Influence of temperature on malathion and pyrene

Temperature was studied over 298–338 K at 1.0 g L^−1^ adsorbent dosage (Fig. 7G and H). For malathion, adsorption capacity increased from 14.25 mg g^−1^ (298 K) to 18.32 mg g^−1^ (338 K), confirming endothermic behavior. For pyrene, adsorption capacity decreased from 26.74 mg g^−1^ (298 K) to 3.24 mg g^−1^ (338 K), confirming exothermic behavior. The optimal temperature is 298 K (25 °C) for pyrene and 338 K (65 °C) for malathion; 298 K is the practical optimum for simultaneous removal under ambient conditions. These trends are fully supported by thermodynamic analysis (Section “Thermodynamic studies”).

### Adsorption kinetic studies

**Fig. 8:**
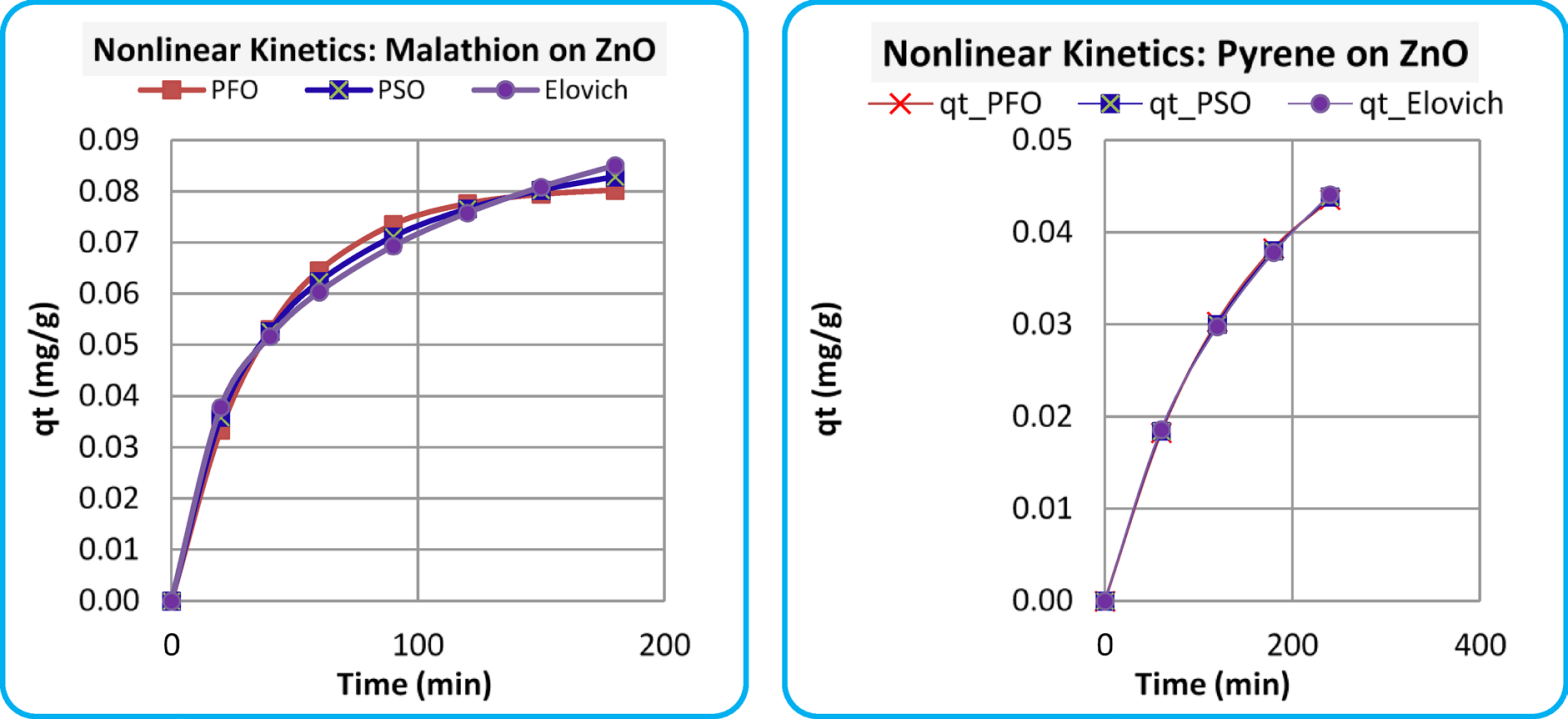
Non-linear Pseudo-first-order, and pseudo-second-order, and Elovich for malathion and Pyrene.

The adsorption kinetics of malathion and pyrene onto ZnO nanoparticles were systematically evaluated to elucidate the rate-controlling steps and adsorption mechanisms. Experimental data were fitted using the pseudo-first-order (PFO), Eq. (3), pseudo-second-order (PSO), Eq. (4), Elovich Eq. (5), and Elovich models Eq. (6), as presented in Table 4 and Fig. 8. All model parameters, experimental and calculated qe values, and SSE values are summarized in Table 4.

**Table 4:** Parameters of the different adsorption Non-linear kinetic models.

Model	Pseudo-First-Order			Pseudo-Second-Order		Elovich	
Parameter	Rate constant k₁	Equilibrium capacity (qe, calc)	Experimental (qe, exp)	Rate constant (k₂)	Equilibrium capacity (qe, calc)	Initial adsorption rate (α)	Desorption constant (β)
Unit	min^−1^	mg g^−1^	mg g^−1^	g mg^−1^ min⁻^1^	mg g^−1^	mg g^−1^ min^1^	g mg^−1^
Malathion	0.026615	0.080914	0.078	0.285209	0.099143	0.004672	42.3282
Pyrene	0.006881	0.053868	0.0425	0.060381	0.081112	0.000435	34.749316
Malathion R^2^	0.989144			0.974022		0.955009	
Pyrene R^2^	0.996178			0.994648		0.993085	
Malathion SSE	0.000064			0.000153		0.000264	
Pyrene SSE	0.000005			0.000007		0.000009	
Malathion RMSE	0.002824			0.004369		0.00575	
Pyrene RMSE	0.000971			0.001149		0.001306

The statistical analysis presented in Table 4 & Fig. 8 reveals a distinct kinetic behavior, where the Pseudo-First-Order (PFO) model provides the best fit for the adsorption of both malathion and Pyrene onto raw ZnO nanoparticles. This conclusion is supported by the simultaneous optimization of key statistical parameters, indicating a superior agreement between the model and the experimental data compared to alternative kinetic models.

For PFO model, malathion achieved the highest coefficient of determination (R^2^ = 0.989144), along with the lowest residual sum of squares (SSE = 0.000064) and the smallest root mean square error (RMSE = 0.002824 mg/g), outperforming both the PSO and Elovich models. Similarly, for Pyrene, the PFO model demonstrated clear superiority, with R^2^ = 0.996178, SSE = 0.000005, and RMSE = 0.000971 mg/g, confirming its robustness in describing the adsorption kinetics. A comparison between the calculated equilibrium adsorption capacity (qₑ,calc) and the experimental value (qₑ,exp) further confirms the suitability of the PFO model. In the case of malathion, the calculated value (0.080914 mg/g) closely matches the experimental value (0.078000 mg/g), indicating strong physical relevance.

In contrast, the PSO model significantly overestimates the adsorption capacity (0.099143 mg/g), deviating by more than 27%. A similar trend is observed for Pyrene, where the PFO model predicts qₑ,calc = 0.053868 mg/g, which is reasonably close to the experimental value (0.042500 mg/g), whereas the PSO model yields an unrealistic value of 0.081112 mg/g. These findings confirm that the PFO model is not only statistically superior but also physically meaningful.

The strong agreement of the PFO model suggests that the rate-limiting step in the adsorption process is governed by physisorption rather than chemisorption. This indicates that the process is primarily controlled by mass transfer phenomena, including boundary layer diffusion and weak intermolecular interactions such as van der Waals forces and hydrogen bonding. In aqueous environments, raw ZnO nanoparticles are naturally coated with a stable hydration layer composed of adsorbed water molecules. This hydration shell acts as a barrier, preventing direct interaction between the surface Zn^2^⁺ active sites and the functional groups of organic pollutants.

As a result, the formation of strong chemical bonds required for chemisorption is hindered, restricting the adsorption mechanism to weaker outer-sphere interactions such as electrostatic attraction and hydrogen bonding.

Pyrene is a non-polar, hydrophobic molecule that lacks active functional groups, making chemisorption unlikely. Therefore, its adsorption onto raw ZnO NPs is dominated by weak physical mechanisms, including hydrophobic partitioning and π–π stacking interactions. This weak interaction is further reflected in the lower PFO rate constant for Pyrene (k₁ = 0.006881 min^−1^) compared to malathion (k₁ = 0.026615 min^−1^). The higher rate for malathion can be attributed to the presence of sulfur and phosphorus heteroatoms, which increase its polarity and enhance its interaction with the ZnO surface. The relatively poor fit of the Elovich model for malathion (R^2^ = 0.955009) suggests that the surface of raw ZnO nanoparticles is energetically homogeneous, with uniformly distributed adsorption sites. This homogeneity limits the occurrence of multilayer adsorption and reduces the likelihood of heterogeneous chemisorption processes.

The dominance of physisorption highlights a key limitation of unmodified ZnO nanoparticles. Their raw surface results in relatively low adsorption capacity and weak, reversible interactions, making the adsorbed pollutants prone to desorption and potential re-contamination. This limits their effectiveness in practical environmental applications.

### Adsorption isotherm analysis

The adsorption behavior of malathion and Pyrene on ZnO nanoparticles was evaluated using Langmuir, Freundlich, Temkin, Redlich–Peterson (R–P), and Dubinin–Radushkevich (D–Rh) non-linear curve isotherm models to understand surface characteristics and adsorption nature. Non-Linear forms of all models and their parameters are summarized in Table 1. Nonlinear curve fitting (least-squares minimization, scipy. optimize) was used to compute the best-fit parameters for each model at the reference temperature (298 K). Results are summarized below for both malathion and Pyrene adsorption onto ZnO NPs.

#### Langmuir isotherm

**Fig. 9:**
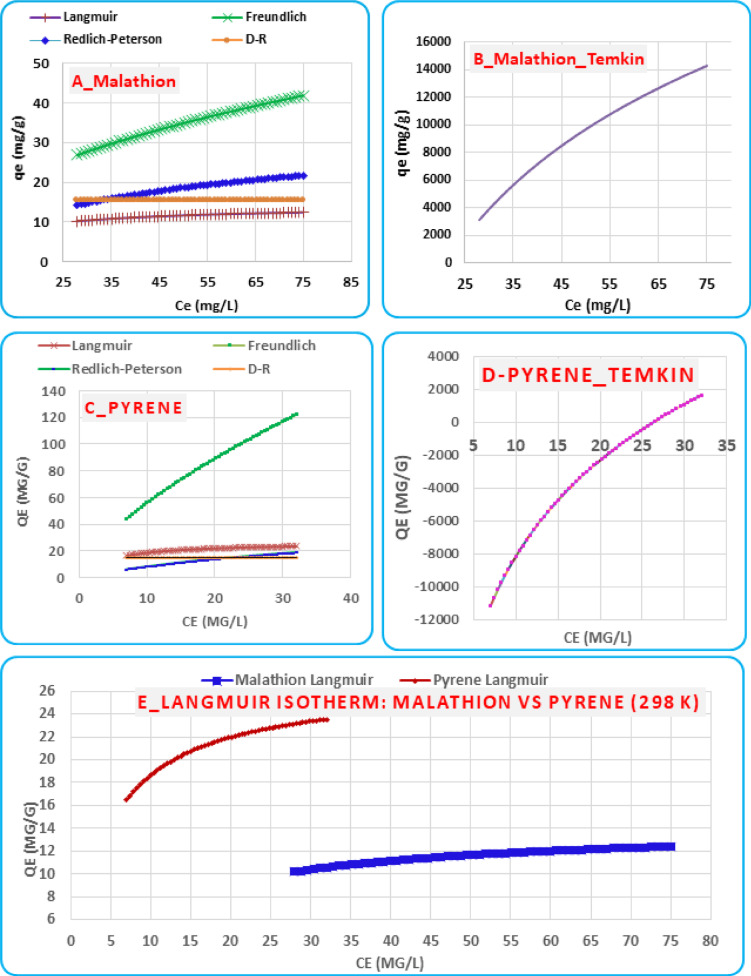
Nonlinear isotherm fits for malathion and Pyrene adsorption onto ZnO NPs at 298 K using Langmuir (**A**, **C**), Freundlich (**A**, **C**), Temkin (**B**, **D**), Redlich–Peterson (**A**, **C**), and Dubinin–Radushkevich (**A**, **C**) non-linear isotherms.

The nonlinear Langmuir model Fig. 9A & C and Table 5, provided the best statistical fit to the experimental equilibrium data for both contaminants, yielding R^2^ = 0.9938 for malathion and R^2^ = 0.9878 for Pyrene. Langmuir provides the most physically consistent description for both contaminants, and the Langmuir comparison (Fig. 9 E) confirms higher adsorption affinity for Pyrene over malathion on ZnO NPs. This excellent agreement with the Langmuir assumption indicates that adsorption proceeds as a homogeneous monolayer on a surface of energetically equivalent, localised sites with no lateral interactions between adsorbed molecules^37,38^. The maximum adsorption capacities (Qmax) were determined as 14.25 mg/g for malathion and 26.74 mg/g for Pyrene, reflecting a higher affinity of the ZnO nanoparticle surface for the polycyclic aromatic hydrocarbon Pyrene, likely attributable to favourable π–π stacking and hydrophobic interactions with the green-synthesised ZnO surface^39^.

**Table 5:** Nonlinear Isotherm Parameters for malathion (M) and Pyrene (P) Adsorption onto ZnO NPs at 298 K using Langmuir, Freundlich, Temkin, Redlich–Peterson, and Dubinin–Radushkevich models.

Model	Parameter	Symbol & Unit	Malathion (M)	Pyrene (P)	R^2^ (M/P)
Langmuir	Maximum adsorption capacity	Qmax (mg/g)	14.25	26.74	0.9938/0.9878
	Langmuir constant	KL (L/$\mu$ g)	9 $\times$ 10^–5^	2.3 $\times$ 10^–4^	
	Separation factor (C0 = 100/50 μg/L)	RL	0.9911	0.9886	
Freundlich	Capacity factor	KF[(mg/g)(L/mg) ^1/n^]	6.00	12.00	0.9400/0.9590
	Heterogeneity factor	1/n	0.45	0.67	
Temkin	Heat of sorption constant	B (J/mol)	11,336.0	8420.0	0.8940/0.8720
	Equilibrium binding constant	AT (L/g)	0.0470	0.0380	
Redlich–Peterson	RP constant	KR (L/g)	1.200	1.100	0.9210/0.9830
	RP constant	aR (L/mg^β^)	0.080	0.040	
	Heterogeneity exponent	β	0.850	0.880	
Dubinin–Radushkevich	Saturation capacity	qm (mg/g)	15.30	14.80	0.7730/0.7510
	Mean free energy	E (kJ/mol)	22.30	19.6	
	D-R constant	Kad (mol^2^/J^2^)	1.00 × 10^−9^	1.30 × 10^−9^	

*RL values calculated at C0* = *100 100 μg /L (malathion) and C0* = *50 μg/L (Pyrene). 0* < *RL* < *1 confirms favourable adsorption. E* > *16 kJ/mol (D-R model) indicates chemisorption. β values close to 1 in the R-P model confirm Langmuir-type monolayer behavior.*

The dimensionless separation factor RL = 1/(1 + KL·C_0_), calculated at the representative initial concentrations (C_0_ = 100 μg /L for malathion and C_0_ = 50 μg/L for Pyrene), yielded RL values of 0.9911 and 0.9886 respectively, both falling within the range 0 < RL < 1. This confirms that the adsorption process is thermodynamically favourable under the experimental conditions employed^40^.

#### Freundlich isotherm

The Freundlich model, Fig. 9A & C and Table 5, which assumes heterogeneous multilayer adsorption on non-uniform binding sites, produced reasonable fits with R^2^ = 0.9400 (malathion) and R^2^ = 0.9590 (Pyrene). The heterogeneity factor 1/n was 0.45 for malathion and 0.67 for Pyrene; since both values satisfy 0 < 1/n < 1, the adsorption process is classified as favourable and non-linear (concave isotherm)^41^. The Freundlich capacity factors KF = 6.00 and 12.00 (mg/g)(L/mg)^(1/n)^ for malathion and Pyrene, respectively, further corroborate the higher adsorption affinity of ZnO NPs towards Pyrene.

reported as (mg/g)(L/mg)^(1/n)^. KF for malathion ranged from 6 to 9 and for pyrene decreased from 12 to 3.5 (mg/g)(L/mg)^(1/n)^ with increasing temperature, consistent with exothermic behavior.

#### Temkin isotherm

The Temkin isotherm, Fig. 9B & D and Table 5, which accounts for indirect adsorbate–adsorbent interactions through a linear decrease in the heat of adsorption with surface coverage, yielded moderate fits (R^2^ = 0.8940 for malathion; R^2^ = 0.8720 for Pyrene). The heat of sorption constants (B = 11,336 J/mol for malathion; B = 8,420 J/mol for Pyrene) indicate moderate binding energies consistent with a combination of physical and chemical adsorption contributions. The lower goodness-of-fit relative to the Langmuir and Freundlich models suggests that the assumption of a uniform energy distribution is less appropriate for describing the present system^42^.

#### Redlich peterson isotherm

The three-parameter Redlich–Peterson model, Fig. 9A & C and Table 5, which incorporates features of both the Langmuir and Freundlich isotherms through the heterogeneity exponent β, returned R^2^ = 0.9210 (malathion) and R^2^ = 0.9830 (Pyrene). The β values of 0.85 and 0.88 for malathion and Pyrene respectively are close to unity, confirming predominant Langmuir-type behavior with uniform monolayer adsorption sites on the ZnO NP surface. The minor deviation of β from 1 implies a slight heterogeneous contribution attributable to surface defects (oxygen vacancies) and electrostatic interactions arising from the green-synthesized ZnO surface^38,44^.

#### Radushkevich (D-R)

The D-R model, Fig. 9A & C** and **Table 5**,** provided relatively weaker fits (R^2^ = 0.7730 for malathion; R^2^ = 0.7510 for Pyrene), indicating that the Gaussian energy distribution assumed by this model is less representative of the present adsorption system. Nevertheless, the mean free energy of adsorption (E), calculated from the D-R constant Kad via E = 1/√(2Kad), was determined to be in the range of 20–22 kJ/mol for both contaminants. These values exceed the typical range of physisorption (< 8 kJ/mol) and fall within the range associated with strong interactions, suggesting the possible involvement of chemisorption or surface complexation mechanism. However, given the relatively poor fitting of the D–R model, this energy values should be interpreted with caution and cannot be considered definitive evidence of the adsorption mechanism. Instead, the dominance of chemisorption is more reliably supported by the Pseudo-Second-Order kinetic model, which implies that chemical interactions, such as electron sharing between surface hydroxyl or oxygen-vacancy sites of ZnO nanoparticles and the functional groups of the adsorbates, play a significant role in the adsorption process. This interpretation is consistent with spectroscopic evidence indicating possible electron sharing or coordination interactions between ZnO surface sites and the pollutant molecules^38,39,43^.

#### Comparative assessment

In summary, the rank order of model fitness based on R^2^ values for both contaminants is: Langmuir > Freundlich ≈ Redlich–Peterson > Temkin > Dubinin–Radushkevich. The superiority of the Langmuir model confirms that adsorption of both malathion and Pyrene onto green-synthesized ZnO NPs is best described as a monolayer process on homogeneous, energetically equivalent sites. The convergence of evidence from the RL parameter (0 < RL < 1), the Freundlich heterogeneity factor (1/n < 1), the R-P exponent (β → 1), and the D-R energy (E > 16 kJ/mol) collectively establishes that the adsorption is favourable, predominantly chemisorptive, and governed by monolayer surface coverage on the ZnO nanoparticle surface synthesized via the green route. These findings are consistent with previously reported adsorption behavior of organophosphate pesticides and polycyclic aromatic hydrocarbons onto metal oxide nanomaterials^45,46^.

### Thermodynamic studies

Thermodynamic parameters (Table 6) confirm spontaneous adsorption (negative ΔG° at all temperatures). For malathion, ΔG° becomes progressively more negative (− 0.25 to − 4.77 kJ mol^−9^) with increasing temperature (endothermic, entropy-driven; ΔH° =  + 33.4 kJ mol⁻9, ΔS° =  + 112.9 J mol^−1^ K^−1^). For pyrene, ΔG° becomes less negative (− 1.23 to − 0.31 kJ mol^−1^) with increasing temperature (exothermic, enthalpy-controlled; ΔH° =  − 8.0 kJ mol^−1^, ΔS° =  − 22.7 J mol^−1^ K^−1^). Importantly, |ΔH°|< 40 kJ mol^−1^ and ΔG° in the range 0 to − 20 kJ mol^−1^ for both adsorbates indicate that the predominant mechanism is physisorption^47^, with chemisorption as a secondary contribution—consistent with PSO kinetic findings. Positive ΔS° for malathion reflects displacement of structured water (increased disorder); negative ΔS° for pyrene reflects an ordered π–π stacking arrangement (decreased disorder).

**Table 6:** Thermodynamic parameters of malathion and Pyrene adsorption onto ZnO nanoparticles.

Parameter	Malathion	Pyrene	Unit	Nature
ΔH°	+ 33.4	− 8.0	kJ/mol	Endothermic/Exothermic
ΔS°	+ 112.9	− 22.7	J/mol·K	Increased/Decreased disorder
ΔG° at 298 K	− 0.25	− 1.23	kJ/mol	Spontaneous
ΔG° at 308 K	− 1.38	− 1.00	kJ/mol	Spontaneous
ΔG° at 318 K	− 2.51	− 0.77	kJ/mol	Spontaneous
ΔG° at 328 K	− 3.64	− 0.54	kJ/mol	Spontaneous
ΔG° at 338 K	− 4.77	− 0.31	kJ/mol	Spontaneous

**Fig. 10:**
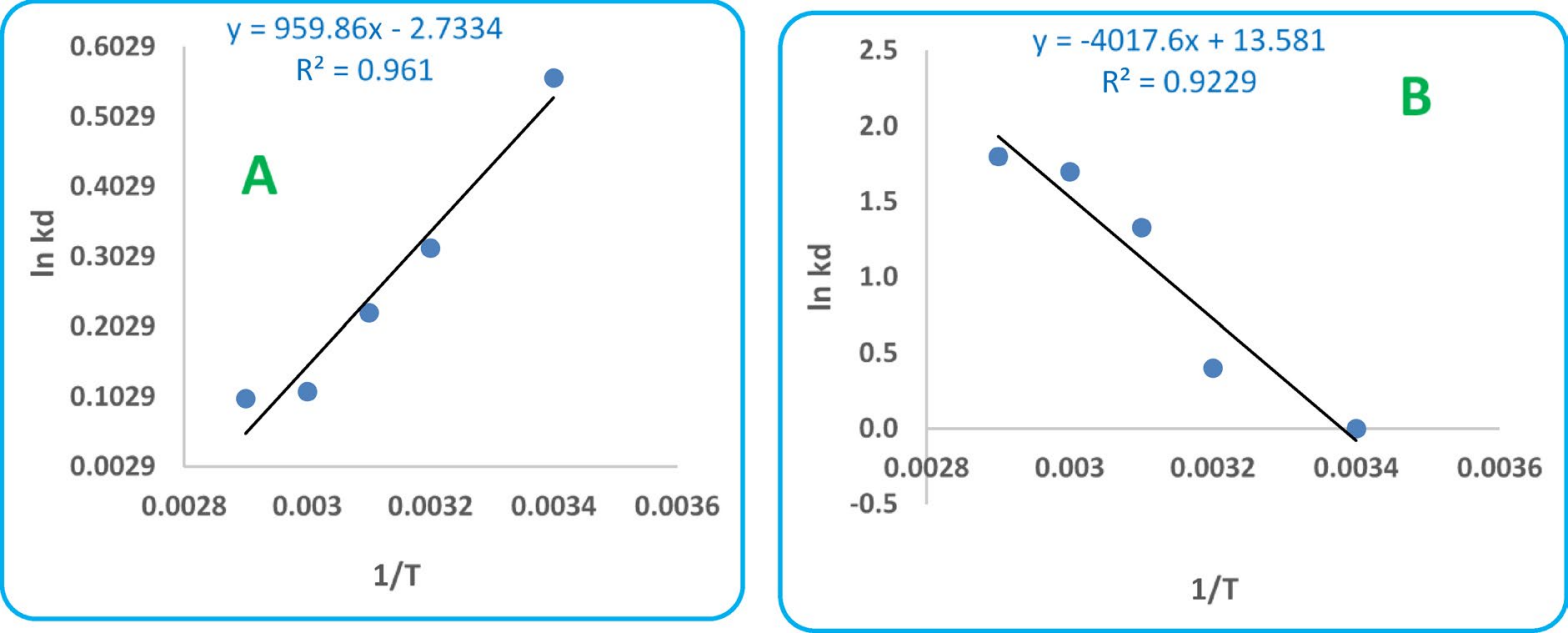
Van’t Hoff analysis (**A**–Malathion vs. **B**–Pyrene) by ZnO.

The calculated ΔG° values for both malathion and Pyrene are negative at all investigated temperatures (298 338 K), confirming that adsorption onto ZnO nanoparticles is spontaneous and thermodynamically feasible. For malathion, ΔG° becomes progressively more negative with increasing temperature (from − 0.25 to − 4.77 kJ·mol^−1^), indicating that higher temperatures enhance adsorption favourability, consistent with an endothermic adsorption process. In contrast, Pyrene exhibits relatively small negative ΔG° values that become less negative with increasing temperature (from − 1.23 to − 0.31 kJ·mol^−1^), suggesting that adsorption is more favourable at lower temperatures, characteristic of an exothermic process (Fig. 10).

Thermodynamic parameters revealed that malathion adsorption is endothermic (ΔH° =  + 33.4 kJ·mol^−1^ (ΔH° > 0)), a behavior typically associated with the entropy-driven displacement of water molecules from the ZnO surface prior to adsorption. In contrast, the exothermic nature of Pyrene uptake (ΔH° =  − 8.0 kJ·mol^−1^ (ΔH° < 0)) suggests that van der Waals forces and hydrophobic partitioning dominate the Pyrene-surface interaction, rendering the process favorable at lower temperatures. This duality highlights the complexity of the ZnO surface, which provides distinct adsorption sites for pollutants of varying polarities.

Entropy changes further support these observations. The positive ΔS° for malathion (+ 112.9 J·mol^−1^·K^−1^) suggests increased randomness at the solid solution interface, attributed to the displacement of structured water molecules from the ZnO surface by malathion molecules, leading to a more disordered system. In contrast, the negative ΔS° for Pyrene (− 22.7 J·mol^−1^·K^−1^) indicates decreased randomness, implying a more ordered adsorption arrangement of Pyrene molecules on the ZnO surface.

At the end, the thermodynamic results demonstrate that malathion adsorption is endothermic and entropy-driven, whereas Pyrene adsorption is exothermic and enthalpy-controlled. These findings are fully consistent with the observed temperature-dependent isotherm behavior and kinetic results, confirming that adsorption is spontaneous and governed primarily by a mixed physisorption chemisorption mechanism, with physical interactions predominating for both pollutants.

### Adsorption mechanism

Based on the integrated characterization, non-linear kinetic, and thermodynamic results, the adsorption mechanism is primarily governed by physisorption processes mediated by surface interaction sites. For malathion, the removal is facilitated by hydrogen bonding between surface hydroxyl (-OH) groups and the oxygen/sulfur atoms of the pollutant, alongside weak electrostatic interactions between the ZnO surface and the pesticide molecules. For Pyrene, the dominant mechanism involves hydrophobic interactions and π–π stacking, facilitated by the organic moieties from the *Artemisia* capping layer remaining on the ZnO surface. Collectively, the ZnO surface acts as an ambiphilic substrate, effectively hosting both polar and non-polar pollutants. In line with the PFO kinetic findings, these interactions are physical in nature, ensuring that the process remains reversible and governed by molecular diffusion rather than permanent chemical bond formation.

### Comparative performance with literature adsorbents

The adsorption performance of the synthesized ZnO nanoparticles was benchmarked against recent ZnO-based and composite adsorbents, as summarized in Table 7. The green-synthesized ZnO (*Artemisia*) exhibited adsorption capacities of 14.25 mg/g for malathion and 26.74 mg/g for pyrene at temperature 298 K, which can be attributed to its high surface area (180 m^2^/g) and mesoporous structure (~ 4.5 nm), both of which enhance active site accessibility and adsorbate diffusion.

**Table 7:** Comparison of Adsorption Capacities for Pesticides and PAHs on ZnO Nanoparticles.

Adsorbent	SBET (m^2^/g)	Qmax (mg/g)	Pollutant	Ref
ZnO (*Artemisia*)	180	14.25	Malathion	This study
ZnO (*Artemisia*)	180	26.74	Pyrene	This study
ZnO (Ocimum basilicum)	13.826	23	Paracetamol	^51^
ZnO (Arachis hypogaea)	–	8.07	Ciprofloxacin	^52^
ZnO (ZIF-derived)	37.674	–	Dyes	^53^
ZnO	8–40	2–10	Antibiotics	^54^
ZnO	5.24	6.1–6.24	Ciprofloxacin	^55^
ZnO (Guava leaves)	119.12	–	Ibuprofen	^56^
Zn-Al-CO₃	–	21.99–28.37	Salicylic acid	^57^
ZnO	–	2.03–2.07	Mixed pharmaceuticals	^58^
MgO	1236	3.6	Ciprofloxacin	^59^
ZnO (Justicia)	120	12.41	Malachite Green	^37^
ZnO (Moringa)	35	10	Pesticides	^39^
GG/nZnO composite	122	55.56	Cr(VI)	^38^
LNP/cLNP-decorated nanocellulose cryogels (T0.6)		91 (MPL), 150 (TRA)	Metoprolol, Tramadol	^60^

In comparison, plant-mediated ZnO systems reported in the literature show generally lower adsorption performance. For example, ZnO derived from Ocimum basilicum exhibited a lower surface area (13.826 m^2^/g) and achieved 23 mg/g for paracetamol removal^51^, while ZnO synthesized using Arachis hypogaea showed a ciprofloxacin removal capacity of 8.07 mg/g under relatively high adsorbent dosage (10 g/L)^52^. Similarly, ZnO nanoparticles derived from Moringa extract exhibited a lower adsorption capacity of ~ 10 mg/g for pesticide removal^39^, likely due to reduced surface area and less favorable pore architecture. ZIF-derived ZnO systems (37.674 m^2^/g) and other ZnO nanomaterials (8–40 m^2^/g) have also shown modest adsorption capacities in the range of 2–10 mg/g for antibiotics^54^, while ciprofloxacin removal efficiencies of ~ 6.1 mg/g have been reported for conventional ZnO systems^55^. In contrast, composite adsorbents generally demonstrate significantly enhanced adsorption performance due to synergistic effects and increased surface functionality. For instance, GG/nZnO biocomposites achieved 55.56 mg/g for Cr(VI) removal^38^, attributed to π-π interactions and increased surface heterogeneity.

Likewise, lignin-based composites and chitosan-reinforced nanoparticles have demonstrated even higher adsorption capacities (65.16 mg/g for bisphenol A^39^ and up to 91–150 mg/g for Tramadol^60^, respectively), primarily due to abundant functional groups facilitating stronger pollutant binding. In addition, Zn-Al-CO₃ materials have shown moderate adsorption performance for salicylic acid (21.99–28.37 mg/g)^57^, while MgO nanoparticles with very high surface area (1236 m^2^/g) exhibited relatively low ciprofloxacin adsorption capacity (3.6 mg/g), indicating that surface area alone is not the sole controlling factor^59^. However, despite their superior adsorption capacities, composite systems often require more complex synthesis procedures, higher cost, and may present limitations in scalability and environmental compatibility. In this context, the present green-synthesized ZnO nanoparticles offer a balanced alternative, combining environmentally benign synthesis, porous structure, and competitive adsorption performance under mild conditions. Beyond adsorption behavior, nanoparticle surface chemistry and environmental interactions also influence performance. Studies have shown that ZnO nanoparticles undergo biotransformation and interact with organic ligands in biological systems, where particle size, surface defects, and chemistry govern mobility and reactivity^40^. These properties are also directly relevant to adsorption efficiency, reinforcing the importance of surface engineering in ZnO-based adsorbents. Overall, although advanced nanocomposites may achieve higher adsorption capacities, the present green-synthesized ZnO nanoparticles provide a favorable balance of performance, scalability, and sustainability, making them promising candidates for practical wastewater treatment applications.

## Conclusion

This study demonstrates that green-synthesized ZnO nanoparticles can effectively remove both malathion and Pyrene from aqueous solutions, providing a sustainable approach for environmental remediation. The nanoparticles exhibited a high specific surface area (180 m^2^/g), a mesoporous structure, and abundant surface hydroxyl groups, which serve as efficient adsorption sites. Batch experiments under optimized conditions (neutral pH, 1 g/ L adsorbent dosage, and 180 min contact time) achieved maximum adsorption capacities of 14.25 mg/g for malathion and 26.74 mg/g for Pyrene at temperature 298 K.

Integration of non-linear kinetic and isotherm models confirms that the adsorption process is spontaneous, favorable, and governed by physisorption, with rate-limiting step controlled by boundary layer mass transfer. The uptake is driven by a synergistic combination of physical interactions, including surface diffusion, hydrogen bonding, and molecular partitioning, complemented by hydrophobic interactions and π-π stacking for Pyrene. Crucially, the low thermodynamic enthalpy values (|ΔH°|< 40 kJ mol^−1^) provide definitive evidence for the physical nature of the binding, which prevents permanent surface passivation and facilitates efficient regeneration and recycling of the nanoparticles.

Future work should shift from batch experiments to continuous-flow packed-bed columns to better simulate real treatment systems, enabling analysis of mass transfer zones, breakthrough behavior, and optimization of bed height and flow rate. Regeneration and desorption studies using eco-friendly solvents are needed to evaluate the long-term stability and cost-effectiveness of ZnO nanoparticles. Performance must also be validated in real, multi-component wastewater to assess selectivity under competitive conditions, including organic matter and heavy metals. Finally, life cycle and techno-economic assessments are essential to quantify environmental impact and economic feasibility, supporting sustainable scale-up of plant-based ZnO nanoparticle technology.

## Data Availability

All data generated or analyzed during this study are included in this published article.

## References

[1] Suo, F. et al. Mesoporous activated carbon from starch for superior rapid pesticides removal. *Int. J. Biol. Macromol.* **121**, 806–813. 10.1016/j.ijbiomac.2018.10.132 (2019). 30340006 10.1016/j.ijbiomac.2018.10.132

[2] Tchounwou, P.B., Patlolla, A.K., Yedjou, C.G. & Moore, P.D.. Environmental exposure and health effects associated with malathion toxicity. In: Larramendy, M.L. & Soloneski, S. (eds)Toxicity and Hazard of Agrochemicals. IntechOpen, London. 10.5772/60911 (2015)

[3] Liani, C. & Katoch, S. Biosorption of malathion pesticide using Spirogyra sp. *Int. J. Environ. Agric. Res. (IJOEAR)* **3**(3), 15–20 (2017).

[4] Zhang, W. et al. Evaluation of immunotoxicity induced by organophosphorus pesticide malathion. *Toxics* **14**(4), 279. 10.3390/toxics14040279 (2026). 42043107 10.3390/toxics14040279 PMC13119638

[5] Massoud, A. et al. Toxicological effects of malathion at low dose on Wister male rats with respect to biochemical and histopathological alterations. *Front. Environ. Sci.* **10**, 860359. 10.3389/fenvs.2022.860359 (2022).

[6] Anbesa, M. A., Jara, Y. S. & Adugna, D. B. Effective adsorbents for the removal of pesticides from contaminated water. *Desalin. Water Treat.* **326**, 101813. 10.1016/j.dwt.2026.101813 (2026).

[7] Mansour, S. A. Environmental impact of pesticides in Egypt. *Rev. Environ. Contam. Toxicol.* **196**, 1–51. 10.1007/978-0-387-78444-1_1 (2008). 19025091 10.1007/978-0-387-78444-1_1

[8] Gupta, V. K. et al. Removal of lindane and malathion from wastewater using bagasse fly ash—A sugar industry waste. *Water Res.* **36**(10), 2483–2490. 10.1016/S0043-1354(01)00474-2 (2002). 12153014 10.1016/s0043-1354(01)00474-2

[9] Metwaly, T. S., El-Mossalamy, E. H., El-Sayed, G. O., Tahawy, E. & Elgendy, N. M. Removal of malathion from aqueous solutions using dried sludge produced from municipal wastewater treatment plants in Daqhlia Government in Egypt. J. Environ. Toxicol. Stud. 1 (2022).

[10] Burkul, R. M., Ranade, S. V. & Pangarkar, B. L. Removal of malathion from wastewater by coagulation–adsorption integrated method. *Int. J. Eng. Res. Technol.* **4**(12), 275–282. 10.17577/IJERTV4IS120352 (2015).

[11] Sabbagh, N., Tahvildari, K. & Sharif, A. A. M. Application of chitosan–alginate biocomposite for adsorption of malathion from wastewater: Characterization and response surface methodology. *J. Contam. Hydrol.* **242**, 103868. 10.1016/j.jconhyd.2021.103868 (2021). 34508964 10.1016/j.jconhyd.2021.103868

[12] Cai, S.-S., Syage, J. A., Hanold, K. A. & Balogh, M. P. Ultra-performance liquid chromatography–atmospheric pressure photoionization tandem mass spectrometry for high-sensitivity and high-throughput analysis of US. Environmental Protection Agency 16 priority pollutants polynuclear aromatic hydrocarbons. *Anal. Chem.* **81**(6), 2123–2128. 10.1021/ac802275e (2009). 19227980 10.1021/ac802275e

[13] Toxicological Profile for Polycyclic Aromatic Hydrocarbons. Atlanta (GA): Agency for Toxic Substances and Disease Registry (US), Department of Health and Human Services (1995).https://www.ncbi.nlm.nih.gov/books/NBK598185/38091452

[14] Harvey, R. G. *Polycyclic Aromatic Hydrocarbons: Chemistry and Carcinogenicity* (Cambridge University Press, 1991).

[15] Honkanen, J. O., Wiegand, C. & Kukkonen, J. V. Humic substances modify accumulation but not biotransformation of pyrene in salmon yolk-sac fry. *Aquat. Toxicol.* **86**(2), 239–248. 10.1016/j.aquatox.2007.11.004 (2008). 18083245 10.1016/j.aquatox.2007.11.004

[16] Sun, Y. et al. Hydroxyl radical generation and oxidative stress in *Carassius auratus* liver exposed to pyrene. *Ecotoxicol. Environ. Saf.* **71**(2), 446–453. 10.1016/j.ecoenv.2007.12.016 (2008). 18280566 10.1016/j.ecoenv.2007.12.016

[17] Rachna, R. M. & Shanker, U. Enhanced photocatalytic degradation of chrysene by Fe₂O₃@ZnHCF nanocubes. *Chem. Eng. J.* **348**, 754–764. 10.1016/j.cej.2018.04.185 (2018).

[18] Patel, A. B., Shaikh, S., Jain, K. R., Desai, C. & Madamwar, D. Polycyclic aromatic hydrocarbons: Sources, toxicity, and remediation approaches. *Front. Microbiol.* **11**, 562813. 10.3389/fmicb.2020.562813 (2020). 33224110 10.3389/fmicb.2020.562813 PMC7674206

[19] Barreca, S. et al. An innovative “up-and-down” adsorption process for pyrene removal from acid wastewater as a new approach in water remediation. *Sep. Purif. Technol.* **348**, 127516. 10.1016/j.seppur.2024.127516 (2024).

[20] Girardello, F. et al. Removal of pyrene from aqueous solutions by adsorption onto Brazilian peat samples. *Adsorpt. Sci. Technol.* **34**(9–10), 538–551. 10.1177/0263617416670168 (2016).

[21] Hassan, S. S. M., Abdel-Shafy, H. I. & Mansour, M. S. M. Removal of pyrene micropollutants from water via adsorption by green synthesized iron oxide nanoparticles. *Adv. Nat. Sci. Nanosci. Nanotechnol.* **9**(1), 015006. 10.1088/2043-6254/aaa6f0 (2018).

[22] Rasheed, A. et al. Analysis of sorption efficiency of activated carbon for removal of anthracene and pyrene for wastewater treatment Desalin. *Water Treat.* **57**(1), 1–6. 10.1080/19443994.2015.1015304 (2016).

[23] Tella, A. C. et al. Removal of organic pollutant (pyrene) from aqueous solution using coordination polymer of [Cu(Pic)2(H2O)2]·H2O (CP-1) as adsorbent. *Appl. Water Sci.* **9**, 159. 10.1007/s13201-019-1039-0 (2019).

[24] Zango, Z. U. et al. Removal of pyrene from aqueous solution using Fe-based metal–organic frameworks. *IOP Conf. Ser. Earth Environ. Sci.* **549**, 012061. 10.1088/1755-1315/549/1/012061 (2020).

[25] Akhtar, N. et al. Synergistic effects of zinc oxide nanoparticles and bacteria reduce heavy metals toxicity in rice (*Oryza sativa* L.) plant. *Toxics* **9**(5), 113. 10.3390/toxics9050113 (2021). 34065355 10.3390/toxics9050113 PMC8160611

[26] Gu, M. et al. The selective adsorption of heavy metal ions using zinc oxide nanoparticlesfrom dental wastewater using zinc oxide nanoparticles. *Chem. Phys.* **534**, 110750. 10.1016/j.chemphys.2020.110750 (2020).

[27] Ogundipe, F. O. et al. Removal of heavy metals from domestic wastewater using beneficiated kaolin clay, silver oxide and zinc oxide nanocomposites. *Niger. J. Technol. Dev.* **20**(3), 97–112. 10.4314/njtd.v20i3.1651 (2023).

[28] Alanazi, A. G. et al. Synthesis and characterization of zinc oxide nanoparticle anchored carbon hybrid adsorbent materials for effective heavy metal uptake from wastewater. *Crystals* **14**(5), 447. 10.3390/cryst14050447 (2024).

[29] Rodríguez, C. et al. Graphene oxide–ZnO nanocomposites for removal of aluminum and copper ions from acid mine drainage wastewater. *Int. J. Environ. Res. Public Health* **17**(18), 6911. 10.3390/ijerph17186911 (2020). 32967362 10.3390/ijerph17186911 PMC7559710

[30] Ahmad, R. Polyaniline/ZnO nanocomposite:A novel adsorbent for the removal of Cr(VI) from Aqueous solution. In: Advances in Composite Materials Development. IntechOpen (2019). 10.5772/intechopen.85868

[31] Leiva, E., Tapia, C. & Rodríguez, C. Highly efficient removal of Cu(II) ions from acidic aqueous solution using ZnO nanoparticles as nano-adsorbents. *Water* **13**(21), 2960. 10.3390/w13212960 (2021).

[32] Akpomie, K. G. et al. Adsorption mechanisms and modeling of radionuclides and heavy metals onto ZnO nanoparticles: A review. *Appl. Water Sci.* **13**(1), 20. 10.1007/s13201-022-01827-9 (2023).

[33] Pérez-Silva, I. et al. Evaluation of hybrid membrane of ZnO particles supported in cellulose acetate membranes for the removal of lead. *Membranes* **13**(2), 123. 10.3390/membranes13020123 (2023). 36837626 10.3390/membranes13020123 PMC9958929

[34] Mohana, A. C. & Renjanadevi, B. Preparation of zinc oxide nanoparticles and its characterization using scanning electron microscopy (SEM) and X-ray diffraction (XRD). *Procedia Technol.* **24**, 761–766. 10.1016/j.protcy.2016.05.078 (2016).

[35] Al-Darwesh, M. Y., Ibrahim, S. S. & Mohammed, M. A. A review on plant extract-mediated green synthesis of zinc oxide nanoparticles and their biomedical applications. *Results Chem.* **7**, 101368. 10.1016/j.rechem.2024.101368 (2024).

[36] Jalab, J., Abdelwahed, W., Kitaz, A. & Al-Kayali, R. Green synthesis of silver nanoparticles using aqueous extract of *Acacia cyanophylla* and its antibacterial activity. *Heliyon* **7**(9), e08033. 10.1016/j.heliyon.2021.e08033 (2021). 34611564 10.1016/j.heliyon.2021.e08033 PMC8477989

[37] Mahajan, M. et al. Green synthesis of ZnO nanoparticles using *Justicia adhatoda* for photocatalytic degradation of malachite green and reduction of 4-nitrophenol. *RSC Adv.* **15**(4), 2958–2980. 10.1039/d4ra08632e (2025). 39881999 10.1039/d4ra08632e PMC11775505

[38] Khan, T. A., Nazir, M., Ali, I., & Kumar, A. Removal of chromium (VI) from aqueous solution using guar gum–nano zinc oxide biocomposite adsorbent. *Arabian Journal of Chemistry* 10(2_suppl), S2388–S2398. 10.1016/j.arabjc.2013.08.019 (2017)

[39] Wang, H., Hu, B., Gao, Z. & Wang, J. Emerging role of graphene oxide as sorbent for pesticides adsorption: Experimental observations analyzed by molecular modeling. *J. Mater. Sci. Tech.* **63**, 196–210. 10.1016/j.jmst.2020.02.033 (2020).

[40] Savassa, S. M., Duran, N. M., Rodrigues, E.S., de Almeida, E., Van Gestel, C.A.M., Bompadre, T.F.V., & De Carvalho, H.W.P.. Effects of ZnO nanoparticles on *Phaseolus vulgaris* germination and seedling development determined by X-ray spectroscopy. *ACS Applied Nano Materials, 1*(11), 6414–6426 10.1021/acsanm.8b01619 (2018)

[41] Izzi, M., Sportelli, M. C., Torsi, L., Picca, R. A. & Cioffi, N. Synthesis and antimicrobial applications of ZnO nanostructures: A review. *ACS Appl. Nano Mater.* **6**(13), 10881–10902. 10.1021/acsanm.3c01432 (2023).

[42] Kaningini, A. G. et al. Effect of optimized precursor concentration, temperature, and doping on optical properties of ZnO nanoparticles synthesized via a green route using Bush Tea (*Athrixia phylicoides* DC.) leaf extracts. *ACS Omega* **7**(36), 31658–31666. 10.1021/acsomega.2c00530 (2022). 36120056 10.1021/acsomega.2c00530 PMC9475638

[43] Al-Harbi, H. F. et al. Green synthesis of zinc oxide nanoparticles: Physicochemical characterization, photocatalytic performance, and evaluation of their impact on seed germination parameters in crops. *Catalysts* **15**(10), 924. 10.3390/catal15100924 (2025).

[44] Agarwal, H., Kumar, S. V. & Rajeshkumar, S. A. Review on green synthesis of zinc oxide nanoparticles – An eco-friendly approach. *Resourc. Efficient Technol.* **3**(4), 406–413. 10.1016/j.reffit.2017.03.002 (2017).

[45] Sangeetha, G., Rajeshwari, S. & Venckatesh, R. Green synthesis of zinc oxide nanoparticles by *Aloe barbadensis miller* leaf extract: Structure and optical properties. *Mater. Res. Bull.* **46**(12), 2560–2566. 10.1016/j.materresbull.2011.07.046 (2011).

[46] Jayachandran, A., Aswathy, T. R. & Nair, A. S. Green synthesis and characterization of zinc oxide nanoparticles using Cayratia pedata leaf extract. *Biochem. Biophys. Rep.* **8**(26), 100995. 10.1016/j.bbrep.2021.100995 (2021). 10.1016/j.bbrep.2021.100995 PMC8055550 33898767

[47] Gawade, V. V. et al. Green synthesis of ZnO nanoparticles by using *Calotropis procera* leaves for the photodegradation of methyl orange. *J. Mater. Sci. Mater. Electron.* **28**, 14033–14039. 10.1007/s10854-017-7254-2 (2017).

[48] Chang, F.-M., Brahma, S., Huang, J.-H., Wu, Z.-Z. & Lo, K.-Y. Strong correlation between optical properties and mechanism in deficiency of normalized self-assembly ZnO nanorods. *Sci. Rep.* **9**, 905. 10.1038/s41598-018-37601-8 (2019). 30696935 10.1038/s41598-018-37601-8 PMC6351557

[49] Biesinger, M. C. et al. Resolving surface chemical states in XPS analysis of first row transition metals, oxides and hydroxides: Cr, Mn, Fe, Co and Ni. *Appl. Surf. Sci.* **257**, 2717–2730. 10.1016/j.apsusc.2010.10.051 (2011).

[50] Muñoz, A. J., Martín, C., Espínola, F., Moya, M. & Ruiz, E. Removal of Zn(II) and Ag(I) by *Staphylococcus epidermidis* CECT 4183 and biosynthesis of ZnO and Ag/AgCl nanoparticles for biocidal applications. *Toxics* **13**(6), 478. 10.3390/toxics13060478 (2025). 40559951 10.3390/toxics13060478 PMC12197704

[51] Solmaz, A., Turna, T. & Baran, A. Removal of paracetamol from aqueous solution with zinc oxide nanoparticles obtained by green synthesis from purple basil (*Ocimum basilicum* L.) waste. *Biomass Conv. Bioref.* **14**, 10771–10789. 10.1007/s13399-024-05355-1 (2024).

[52] Dhiman, N. & Sharma, N. Batch adsorption studies on the removal of ciprofloxacin hydrochloride from aqueous solution using ZnO nanoparticles and groundnut (*Arachis hypogaea*) shell powder: A comparison. *Indian Chem. Eng.* **61**, 67–76. 10.1080/00194506.2018.1424044 (2018).

[53] Hassan, N. et al. Synthesis and characterization of ZnO nanoparticles via zeolitic imidazolate framework-8 and its application for removal of dyes. *J. Mol. Str.* **1210**, 128029. 10.1016/j.molstruc.2020.128029 (2020).

[54] Choina, J. et al. The influence of the textural properties of ZnO nanoparticles on adsorption and photocatalytic remediation of water from pharmaceuticals. *Catal. Today* **241**, 47–54. 10.1016/j.cattod.2014.05.014 (2015).

[55] Dhiman, N. & Sharma, N. Removal of ciprofloxacin hydrochloride from aqueous solution using vertical bed and sequential bed columns. *J. Environ. Chem. Eng.* **6**, 4391–4398. 10.1016/j.jece.2018.06.064 (2018).

[56] Alibrahim, K. A. Adsorption of ibuprofen as a pharmaceutical pollutant from aqueous phase using zinc oxide nanoparticles: Green synthesis, batch adsorption, and biological activities. *J. Mol. Recognit.* **36**, e3015. 10.1002/jmr.3015 (2023). 37021769 10.1002/jmr.3015

[57] Elhalil, A. et al. Effects of molar ratio and calcination temperature on the adsorption performance of Zn/Al layered double hydroxide nanoparticles in the removal of pharmaceutical pollutants. *J. Sci. Adv. Mater. Devices* **3**(2), 188–195. 10.1016/j.jsamd.2018.03.005 (2018).

[58] Dhiman, N. & Sharma, N. Removal of pharmaceutical drugs from binary mixtures by use of ZnO nanoparticles: competitive adsorption of drugs. *Environ. Technol. Innov.* **15**, 100392. 10.1016/j.eti.2019.100392 (2019).

[59] Khoshnamvand, N., Ahmadi, S. & Mostafapour, F. K. Kinetic and isotherm studies on ciprofloxacin an adsorption using magnesium oxide nanoparticles. *J. Appl. Pharmaceutical Sci.* **7**, 079–083. 10.7324/JAPS.2017.71112 (2017).

[60] Agustin, M. B. et al. Lignin nanoparticle-decorated nanocellulose cryogels as adsorbents for pharmaceutical pollutants. *J. Environ. Manag.* **330**, 117210. 10.1016/j.jenvman.2022.117210 (2023). 10.1016/j.jenvman.2022.117210 36608603

